# Pyrolysis Enzymolysis-Treated Pomelo Peel: Porous Carbon Materials with Fe−N_x_ Sites for High-Performance Supercapacitor and Efficient Oxygen Reduction Applications

**DOI:** 10.3390/polym15193879

**Published:** 2023-09-25

**Authors:** Xiangyu Chen, Jiahua Ma, Xiaoshuai Sun, Chuanshan Zhao, Jiehua Li, Hui Li

**Affiliations:** State Key Laboratory of Biobased Material and Green Papermaking, Qilu University of Technology, Shandong Academy of Sciences, Jinan 250353, China; colacxy@163.com (X.C.); 13290394238@163.com (J.M.); 17861407148@163.com (X.S.); 13695310529@163.com (J.L.); 15668434088@163.com (H.L.)

**Keywords:** pomelo peel, enzymatic treatment, Fe−N_x_, supercapacitors, ORR

## Abstract

This paper proposes a different strategy for deriving carbon materials from biomass, abandoning traditional strong corrosive activators and using a top−down approach with a mild green enzyme targeted to degrade the pectin matrix in the inner layer of pomelo peel cotton wool, inducing a large number of nanopores on its surface. Meanwhile, the additional hydrophilic groups produced via an enzymatic treatment can be used to effectively anchor the metallic iron atoms and prepare porous carbon with uniformly dispersed Fe−N_x_ structures, in this case optimizing sample PPE−FeNPC−900’s specific surface area by up to 1435 m^2^ g^−1^. PPE−FeNPC−900 is used as the electrode material in a 6 M KOH electrolyte; it manifests a decent specific capacitance of 400 F g^−1^. The assembled symmetrical supercapacitor exhibits a high energy density of 12.8 Wh kg^−1^ at a 300 W kg^−1^ power density and excellent cycle stability. As a catalyst, it also exhibits a half−wave potential of 0.850 V (vs. RHE) and a diffusion-limited current of 5.79 mA cm^−2^ at 0.3 V (vs. RHE). It has a higher electron transfer number and a lower hydrogen peroxide yield compared to commercial Pt/C catalysts. The green, simple, and efficient strategy designed in this study converts abundant, low−cost waste biomass into high-value multifunctional carbon materials, which are critical for achieving multifunctional applications.

## 1. Introduction

As the global economy and population continue to grow, the global energy demand is increasing at an unprecedented rate. However, traditional fossil fuels are unable to meet this demand, necessitating the development of new energy storage and utilization technologies that are environmentally friendly, economically efficient, and high-performing. Among these technologies, supercapacitors, in particular, have shown excellent charge–discharge capabilities and outstanding stability [1,2,3]. Metal–air cells and fuel cells have also garnered significant attention due to their promising features, such as high power density, stability, and low cost [4,5,6]. The oxygen reduction reaction (ORR) is crucial to the performance of devices such as metal–air cells and fuel cells [7,8,9]. However, the cathodic oxygen reduction reactions in metal–air cells and fuel cells typically rely on expensive Pt−based catalysts with low storage capacity and poor durability, limiting their application [10,11]. Therefore, there is a growing interest in identifying new carbon materials that are abundant, stable, inexpensive, and suitable for both supercapacitors and oxygen reduction reaction catalysts. 

Porous carbon materials have a high specific surface area (SSA) and high porosity with adjustable pore structure layering, good electrical conductivity, and stable physical and chemical properties, making them ideal for use as supercapacitor electrodes and ORR catalysts [12]. Recently, the utilization of natural biomass to prepare porous carbon materials has gained significant attention due to this material’s abundance, low cost, and unique porous structure, including cotton, coconut fiber, chestnut, etc. [13,14,15]. Pomelo peels are an abundant, easily accessible, and renewable resource and can be used as a high−quality biomass carbon source due to their richness in soluble and insoluble compounds. Meanwhile, the white cotton wool inner layer of the pomelo peel has a unique fluffy and porous foam−like structure and abundant oxygen−containing functional groups, making it an ideal biomass feedstock for the preparation of porous carbon applied in supercapacitors and ORR catalysts. However, 2–2.5 million tons of pomelo peel are accumulated as waste in China annually, which poses environmental challenges [16]. Therefore, using pomelo peels to prepare porous biomass carbon materials for multifunctional applications offers an ideal solution for mitigating environmental pollution and avoiding resource waste. 

Previous studies have employed large amounts of strong corrosive chemicals (e.g., potassium hydroxide as a template) to treat pomelo peels for application in SCs, resulting in environmental pollution and impeding practical production [17,18,19,20]. Additionally, the biomass carbon prepared in this manner commonly exhibits a relatively simple structure and lacks surface active sites, rendering it unsuitable for use as an ORR catalyst for metal–air cell and fuel cell reactions [21,22]. Therefore, it is crucial to develop a simpler, more efficient, and environmentally friendly method for preparing multifunctional carbon materials with both exceptional capacitive and ORR performance, which can ultimately advance the development of efficient supercapacitors and ORRs.

In this study, a novel approach was developed to produce efficient and versatile porous carbon materials without using harsh chemical reagents. Instead, the gentle treatment of pectin, a substance abundant in pomelo peel cell walls, with the help of a specific enzyme was carried out. This enzymatic process, known as enzymolysis, effectively broke down the pectin, creating numerous nanopores on the material’s surface. This resulted in an increased surface area and more active sites, ideal for further modifications. After this, Fe and N were doped onto the porous carbon to create an Fe−N_x_ structure with atomic dispersion anchored on the surface, and the best samples had a high SSA of 1453 m^2^ g^−1^. The structure and electrochemical performance were thoroughly characterized as supercapacitor electrodes and ORR catalysts.

## 2. Experimental Section

### 2.1. Materials

Pomelo peel was purchased from a local market in Jinan, Shandong Province, China. Pectinase was purchased from Xia Sheng enzyme retail store. Potassium hydroxide (KOH, electronic grade, 99.99%), ferric chloride hexahydrate (FeCl_3_·6H_2_O), and ammonium chloride (NH_4_Cl, AR, 99.5%) were purchased from Shanghai Aladdin Biochemical Technology Co., Ltd., Shanghai, China.

### 2.2. Preparation of Carbon Precursors via Enzymatic Treatment of Pomelo Peel

The fresh pomelo peels were prepared by removing their yellow skin and cutting their white cotton wool inner layer into 3 × 1.5 × 1 cm^3^ pieces. These pomelo peel pieces were then washed, dried in an oven, and kept aside for later use. To initiate the process, around 3.5 g of dried pomelo peel was mixed with 150 mL of McIlvaine buffer (pH = 4) along with 75 μL and 150 μL of pectinase (with an enzyme activity unit of 80,000 U mL^−1^) and kept shaking at a constant temperature of 50 °C for 12 h, 18 h, and 24 h. Post enzymatic treatment, the pomelo peels were washed with deionized water and then dried in an oven at 60 °C. These treated peels were heated under nitrogen in a tube furnace at a heating rate of 5 °C·min^−1^ to 800 °C for 2 h and identified as PPC−Ex−y, where x represents the amount of pectinase and y represents the time of the enzymatic treatment. As control samples, pomelo peels that did not undergo enzymatic treatment were directly carbonized and named PPC.

### 2.3. Preparation of Hierarchical Porous Fe, N−Doped Carbon Materials

The PPC−E150−18 carbon precursor was selected to prepare Fe, N−doped carbon materials. Firstly, 1.0 g of PPC−E150−18 and 0.5 g of FeCl_3_·6H_2_O were dispersed in 50 mL of deionized water under sonication. The suspension was further dried in an oven at 80 °C. The dried sample was collected, ground together with 20 g of NH_4_Cl, and then subjected to various carbonation temperatures (800 °C, 900 °C, and 1000 °C) in a nitrogen atmosphere at a heating rate of 5 °C·min^−1^ for a period of 2 h. The resulting black solid was washed with 1.0 M of hydrochloric acid for 24 h to wash away the iron oxide within it and then washed with deionized water. The final product was named PPE−FeNPC−T, where T represents the pyrolysis temperature, and it was dried at 60 °C overnight.

To investigate the impact of enzymatic treatment and the role of Fe atom doping, different comparison samples were prepared from PPC or PPC−E150−18 as carbon precursors for comparison with the optimal sample PPE−FeNPC−900. These included PPE−PC−900, which was obtained via the direct carbonization of PPC−E150−18; PPE−NPC−900, which was obtained from PPC−E150−18 without Fe atom doping; and PP−FeNPC−900, which was obtained from PPC without enzymatic treatment.

### 2.4. Electrochemical Measurement

All electrochemical performances were studied using a CHI 760E electrochemical workstation. The details on the procedures used for the preparing electrodes and conducting electrochemical measurements for supercapacitors’ application and use as the catalyst for ORR are provided in the Supporting Information.

## 3. Results and Discussion

### 3.1. Transformation of Pomelo Peel into PPE−FeNPC

**Scheme 1:**
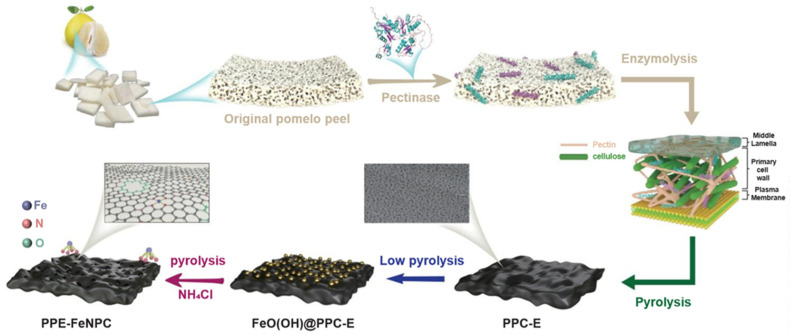
Preparation of pomelo peel−based Fe, N co−doped multifunctional carbon materials based on enzymatic treatment followed by pyrolysis.

As shown in Scheme 1, the proposed green approach for the bulk transformation of pomelo peel involved the selective hydrolysis of insoluble protopectin into water−soluble pectin via an enzyme. This process created numerous shallow nanopores on the surface; then, pyrolysis was carried out to transform the bulk pomelo peel into hierarchically porous carbon materials. Initially, the enzymatic process was initiated by introducing pectinase into the pectin−rich pomelo peel. Pectinase is a complex enzyme system composed of various enzyme types, primarily including four components: protopectinase, polygalacturonase, laccase, and pectin esterase (EC 3.1.1.11). These enzymes work synergistically to break down pectin. The mechanism and effect of enzymatic digestion are depicted in Figure S1. It was found that by degrading pectin, not only more hydrophilic groups were provided but also the surface of the pyrolyzed pomelo peel became rougher and formed a dense pore structure, which not only improved the SSA and porosity of the carbon material but also provided the possibility of exposing more active sites. The addition of FeCl_3_·6H_2_O to the resulting carbon precursor (PPC−E) suspension was followed by low−temperature pyrolysis. During this process, the abundant hydrophilic groups (e.g., −COOH and −OH) of PPC−E coordinated with iron ions [23], and the excess Fe^3+^ in the aqueous solution was hydrolyzed to amorphous Fe(OH)_3_ and FeO(OH) crystals (Equations (1) and (2), and Figure S2), which were deposited on the carbon precursor. During the high−temperature pyrolysis with the introduction of NH_4_Cl, the FeO(OH) crystals in FeO(OH)@PPC−E were initially converted into Fe_2_O_3_ (Equation (3)), and when the temperature was >700 °C, the reducing substances (e.g., H_2_, CO, and graphite carbon) produced during the pyrolysis of pomelo peel reduced Fe_2_O_3_ to Fe_3_O_4_ or further to metallic Fe, which subsequently reacted with volatile HCl to form volatile FeCl_3_ and promoted the decomposition of NH_4_Cl (Equations (4)–(7)). The coordinated iron atoms on the carbon precursor were immobilized by the N species generated from NH_4_Cl’s decomposition, forming Fe−N−C. FeCl_3_·6H_2_O and NH_4_Cl also contributed to the creation of specific microporous and mesoporous structures in the resulting carbon material [24,25]. Finally, acid washing was used to remove iron oxide and undesirable inactive species, resulting in the formation of atomically dispersed iron sites anchored to the N−doped porous carbon material. This process involved the following reactions [25]:

(1) $${\mathrm{Fe}}^{3+}+3H_{2}O\;\to {\;\mathrm{Fe}(\mathrm{OH})}_{3}+3H^{+}$$

(2) $${\mathrm{Fe}(\mathrm{OH})}_{3}\to \mathrm{FeO}\left(\mathrm{OH}\right)+{\;H}_{2}O\;\text{↑}$$

(3) $$2\mathrm{FeO}\left(\mathrm{OH}\right)\to {\;\mathrm{Fe}}_{2}O_{3}+{\;H}_{2}O\;\text{↑}$$

(4) $$3{\mathrm{Fe}}_{2}O_{3}+4H_{2}\left(\mathrm{CO},C\right)\to \;2{\mathrm{Fe}}_{3}O_{4}+4H_{2}O({\mathrm{CO}}_{2},\;\mathrm{CO})\;\text{↑}$$

(5) $${\mathrm{Fe}}_{3}O_{4}+4C\;\to \;3\mathrm{Fe}+4\mathrm{CO}\;\text{↑}$$

(6) $${\mathrm{NH}}_{4}\mathrm{Cl}\to {\mathrm{NH}}_{3}\text{↑}+\mathrm{HCl}\text{↑}$$

(7) $${\mathrm{Fe}}_{3}O_{4}\;(\mathrm{Fe})+6\mathrm{HCl}\text{↑}\to \;2\mathrm{Fe}{\mathrm{Cl}}_{3}\text{↑}+3H_{2}O\text{↑}$$

### 3.2. Structural Characterization and Analysis 

#### 3.2.1. Morphological Characterization 

**Figure 1:**
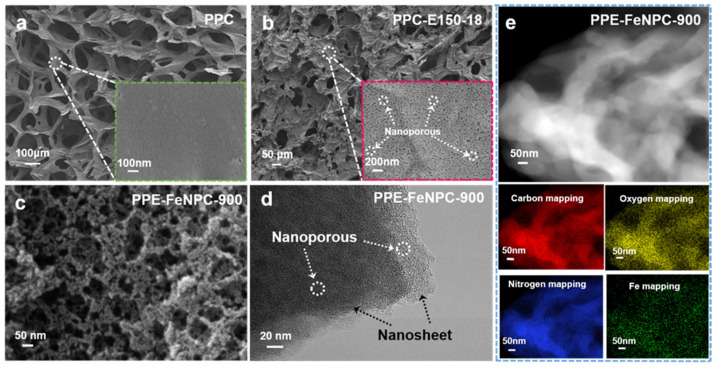
SEM images of (**a**) raw pomelo peel after carbonization; (**b**) enzyme−treated pomelo peel for which an enzyme dose of 150 μL was applied for a period of 18 h; (**c**) sample of PPE−FeNPC−900; (**d**) TEM image of PPE−FeNPC−900; and (**e**) the corresponding element maps showing the distributions of C (red), O (yellow), N (blue), and Fe (green).

The morphology of the original pomelo peel is shown in Figure 1a and Figure S3a,b. An interwoven porous morphology with a smooth, multi−folded shape was observed. This morphology ensured the uniform distribution of enzymes during enzymatic treatment. Although a certain amount of natural orifice structure existed within the pomelo peel, which could somewhat accelerate the ion transfer rate during energy storage, there was still an overall lack of pore structure in the original peel. In contrast, the enzymatically treated pomelo peel exhibited a disrupted sheet−like, stacked morphology (Figure 1b and Figure S3c–l). A large number of nanopore structures of varying sizes were observed on the surface, which not only enhanced charge storage and rapid electrolyte transport, providing high specific capacitance and high power for the capacitor, but also exposed more ORR catalytic active sites [26]. The effects of enzyme dose and disintegration time on the surfaces of different carbon precursors were investigated on samples including PPC−E75−12, PPC−E75−18, PPC−E75−24, PPC−E150−12, PPC−E150−18, and PPC−E150−24. It was found that as the pectinase dosage and degradation time increased, the SSA increased, the surface became rougher, and shallower nanopores appeared (Figures S3 and S4). The pore sizes for all the samples were mainly concentrated in the range of 3–4 nm. Among all the samples, the SSA for samples PPC−E75−24 and PPC−E150−18 was 797.8 and 822.8 m^2^ g^−1^, respectively, with PPC−E150−18 exhibiting a larger SSA. Additionally, severe structural damage occurred when the enzyme dosage was 150 μL and the enzymatic treatment time was 24 h (Figure S5), resulting in a significant reduction in the volume and structural integrity of the peels. After Fe, N co−doping and further pyrolysis at 900 °C, the surface of sample PPC−E150−18 became even rougher, and its porosity increased (Figure 1c). TEM images revealed a nanosheet morphology with a porous structure, which provided a large SSA, dense pores, and efficient ion transport channels (Figure 1d). The elemental map confirmed the uniform distribution of C, O, N, and Fe (Figure 1e), which enhanced the capacitive properties and electrocatalytic activity of the sample.

#### 3.2.2. Structure Analysis

**Figure 2:**
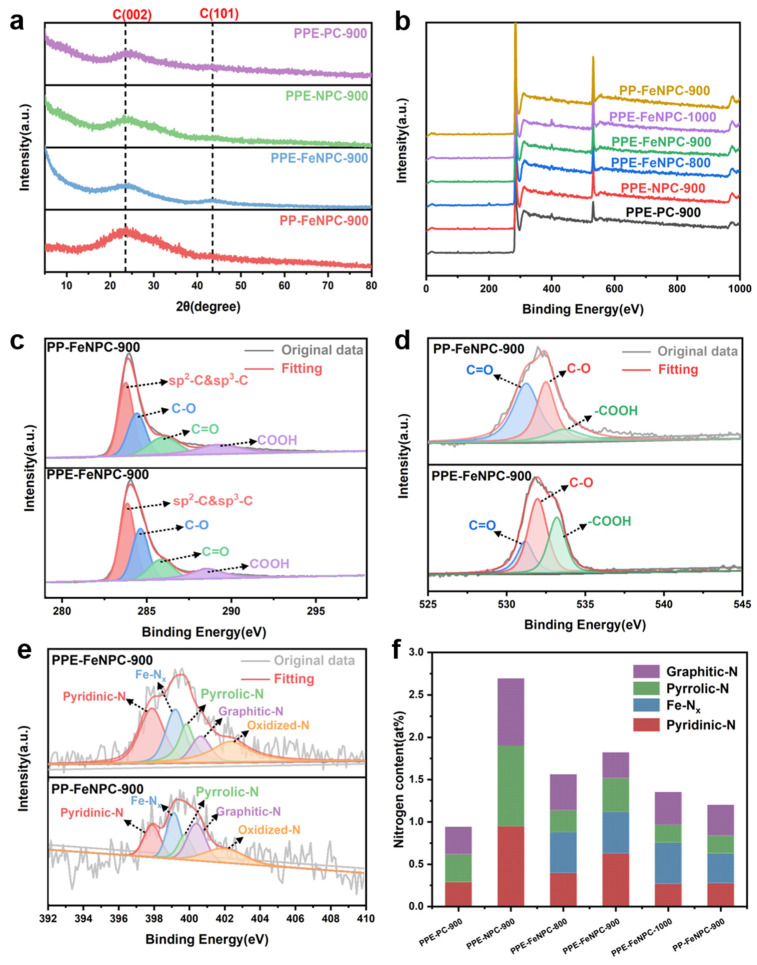
(**a**) XRD patterns of sample PPE−PC−900, PPE−NPC−900, PPE−FeNPC−900, and PP−FeNPC−900; (**b**) XPS survey spectra of all samples; (**c**) XPS C 1s spectra of PP−FeNPC−900 and PPE−FeNPC−900; (**d**) XPS O 1s spectra of PP−FeNPC−900 and PPE−FeNPC−900; (**e**) XPS N 1s spectra of PP−FeNPC−900 and PPE−FeNPC−900; (**f**) N content and configurations of all samples.

The XRD patterns of the PPE−FeNPC−T series were compared before and after hydrochloric acid washing (Figure S6). The XRD pattern of PPE−FeNPC−800 (Figure S6a) showed peaks at 2θ = 24.1°, 33.2°, 35.6°, 39.3°, 49.5°, 54.1°, 57.6°, 62.4°, and 64.0°, which were attributed to the Fe_2_O_3_ lattice plane (PDF#33−0664). Additionally, the XRD pattern also displayed peaks at 2θ = 30.1° and 43.1°, which were attributed to the Fe_3_O_4_ lattice plane (PDF#19−0629). These indicated that the FeO(OH) deposited on the carbon precursor was converted into Fe_2_O_3_ below 800 °C, and some of the Fe_2_O_3_ was reduced to Fe_3_O_4_ via pyrolysis. For sample PPE−FeNPC−900, the number of Fe_2_O_3_ (PDF#33−0664) diffraction peaks decreased, accompanied by an increasing number of Fe_3_O_4_ (PDF#19−0629) diffraction peaks, suggesting a further pyrolytic reduction of Fe_2_O_3_ to Fe_3_O_4_. At 1000 °C, the Fe_2_O_3_ lattice plane disappeared, and the number of Fe_3_O_4_ lattice plane peaks was similarly small, indicating the complete pyrolytic reduction of Fe_2_O_3_ to Fe_3_O_4_ and Fe_3_O_4_ to other amorphous substances. The disappearance of diffraction peaks of Fe_2_O_3_ and Fe_3_O_4_ might also indicate that acid washing was effective in removing Fe_2_O_3_ and Fe_3_O_4_ (Figure S6b). The XRD patterns of the whole series of PPE−FeNPC−T samples (Figure 2a) displayed two broad peaks at 22° and 44°, corresponding to the (002) and (101) crystal planes in carbon, respectively. This indicated all the samples had a disordered and porous carbon structure. Moreover, a large increasing intensity in the low−angle scatter of PPE−FeNPC−T is noted in Figure 2a and Figure S6b, indicative of a highly developed nanoporous structure, which was advantageous for the effective adsorption of electrolyte ions at the electrode interface, consequently leading to enhanced electric double−layer capacitance [27,28].

XPS was employed to determine the binding states of atoms in the carbon structure. Figure 2b displayed the high−resolution spectra of the elements C, N, O, and Fe with binding energies of 285, 400, 533 and 711.4, and 724.4 eV [29,30]. The C 1s spectra of all the samples were deconvoluted into four peaks at 284.1, 284.9, 286.3, and 288.8 eV (Figure 2c and Figure S7), representing sp2/sp3−C, ether and phenol (C−O), quinone (C=O) groups, and carboxyl groups (−COOH), respectively [27]. The O 1s spectra of all the samples were divided into three peaks at 531.4, 532.7, and 534.3 eV (Figure 2d and Figure S8), representing C=O, C−O, and −COOH groups [31]. As shown in Figure 2d, the pectinase−treated PPE−FeNPC−900 exhibited more C−O and −COOH groups compared to PP−FeNPC−900, which revealed that the enzymatically treated pomelo peel presented more methanol and carboxy groups, providing more oxygen−containing functional groups. The doping of O could effectively enhance the wettability of the electrode material and increase its pseudocapacitive response, thereby improving the capacitive properties of the material [32]. The Fe 2p spectra of the PP−FeNPC−900 and PPE−FeNPC−T series of samples (Figure S9) displayed Fe−N signals at 711.4 and 724.4 eV [33], indicating the existence of Fe−N_x_ coordination. No metallic Fe signals were present in any sample. The N 1s spectra of all the samples were divided into five peaks at 398.1, 399.5, 400.4, 400.9, and 401.7 eV (Figure 2e and Figure S10), representing pyridine nitrogen, Fe−N_x_, pyrrole nitrogen, graphite nitrogen, and nitrogen oxide, respectively [34]. The content of Fe−N_x_ increased significantly after pectinase treatment, suggesting that enzymatic treatment contributes to the formation of Fe−N_x_ structures (Figure 2e). Table S1 summarizes the nitrogen content of all the samples in various configurations. It indicates that among all the Fe−N co−doped samples, PPE−FeNPC−900 exhibited the highest total nitrogen and iron content, with 2.21 and 0.43 at%. Additionally, an inductively coupled plasma optical emission spectrometry (ICP−OES) analysis showed that the iron content was ~0.90 wt%. Additionally, PPE−FeNPC−900 displayed the highest levels of pyridine nitrogen, Fe−N_x_, and pyrrole nitrogen (Table S1 and Figure 2f), corresponding to 0.63, 0.49, and 0.4 at%, respectively, as well as graphite nitrogen content, corresponding to 0.3 at%. The presence of Fe−N_x_ positively influenced the capacitive performance of SCs [35], while pyridine and pyrrole nitrogen could generate pseudocapacitance [36], and graphite nitrogen could enhance the capacitive performance and reduce electron transfer resistance by altering the charge density [37]. Furthermore, the carbon atoms adjacent to the pyridine nitrogen served as active sites for O_2_ adsorption, which was the first step in this ORR. Thus, the samples with high pyridine and graphite nitrogen content could lower the energy barrier and thus improve ORR performance. Moreover, the synergistic effect of Fe−N−C, which contributed to the enhancement of ORR activity, exhibited better catalytic performance than the N−doped catalyst table.

**Figure 3:**
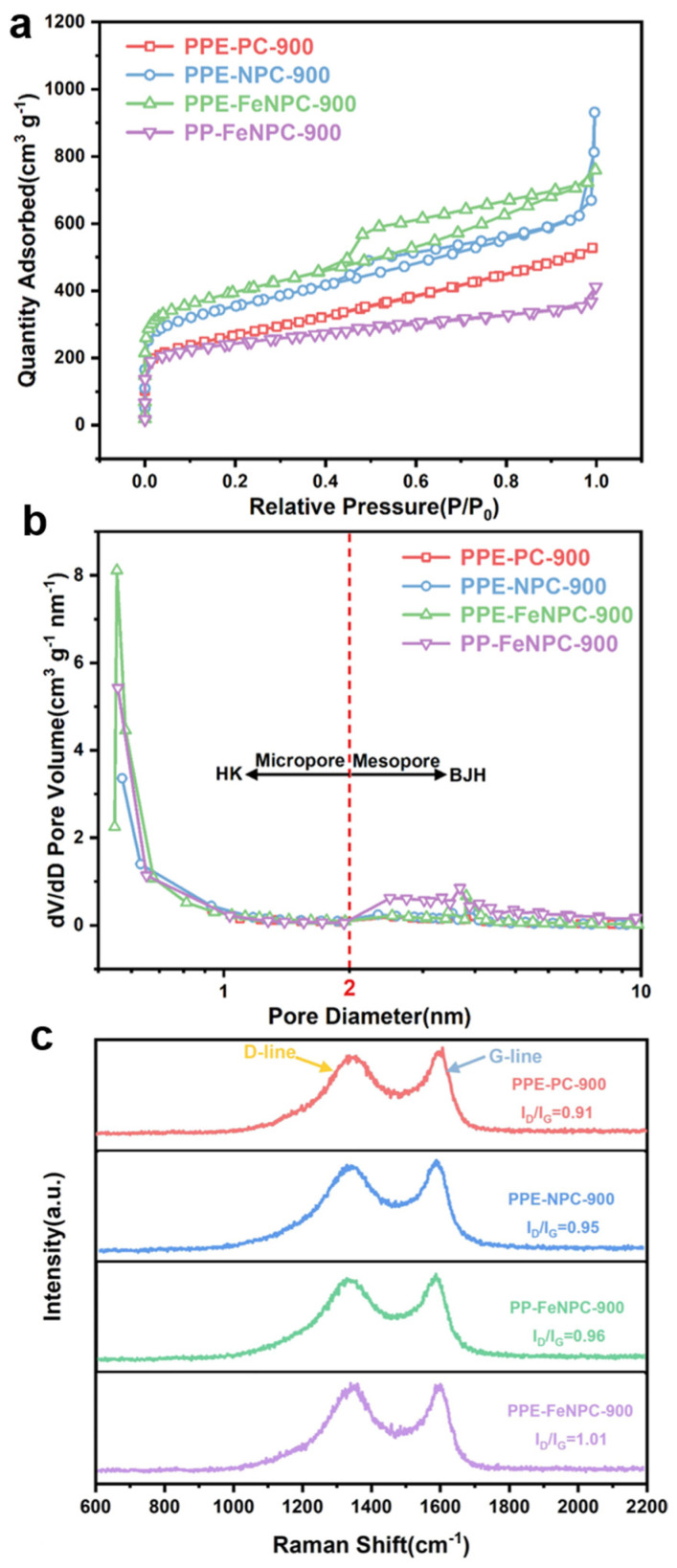
(**a**) N_2_ adsorption and desorption curves of different comparison samples; (**b**) pore size distribution of different comparison samples calculated using the HK method (pore width < 2 nm) and BJH method (pore width ≥ 2 nm); (**c**) Raman spectra of different comparison samples.

Nitrogen adsorption and desorption isotherms were employed to quantify the pore structures of the samples. BET analysis revealed the coexistence of type I and IV isotherms in all the samples (Figure 3a and Figure S11a). The curve increased dramatically at low pressure (P/P_0_ < 0.01), indicating the presence of micropores in the samples, and a hysteresis loop in the high−pressure region (P/P_0_ = 0.4–1.0) signified the presence of mesopores in the samples [38,39]. The detailed textural parameters, including SSA and pore volume, for all the samples are summarized in Table S2. As the temperature increased from 800 °C to 900 °C, the average pore size decreased from 3.4 nm to 3.28 nm, while the pore volume improved significantly, particularly the micropore volume. This observation suggests that the increased presence of Fe−N_x_ structures (in agreement with the XPS results) contributed to the development of additional micropores. The pore structure, primarily characterized by these newly formed micropores, accounted for the slight reduction in average pore size. When the pyrolysis temperature increased to 1000 °C, the average pore size decreased to 2.92 nm, and the mesopore volume declined significantly, suggesting that an excessively high pyrolysis temperature led to the collapse of the pore structure. PPE−FeNPC−900 exhibited the largest SSA and the optimal micropore and mesopore structures, indicating that 900 °C was the optimal pyrolysis temperature. It was also observed that fewer micropores were created in sample PP−FeNPC−900 than in sample PPE−PC−900, revealing that NH_4_Cl and FeCl_3_·6H_2_O could play an activating role during the pyrolysis process, but they were less effective than enzymatic treatment. When comparing samples PPE−NPC−900 and PPE−FeNPC−900, it was found that introducing FeCl_3_·6H_2_O not only significantly increased the SSA of the samples but also enhanced their micropore volume and reduced their average pore size. This may be attributed to the formation of the Fe−N_x_ structure, which could prevent the collapse of micropores to mesopores due to high−temperature pyrolysis. It can be concluded that enzymatic treatment, NH_4_Cl, and FeCl_3_·6H_2_O pyrolysis were essential factors in creating the unique surface structure and high SSA of pomelo−peel−based porous carbon materials. The pore size distribution (Figure 3b and Figure S11b) corroborated our earlier SEM findings that PPE−FeNPC−900 retained enzymatic nanopores, which exposed more catalytic active sites in close contact with oxygen molecules, as well as a uniformly dispersed Fe−N_x_ structure, which led to exceptional ORR performance. As micropores are the active sites of ion storage, their formation provides more favorable conditions for electrolyte adsorption. It can be seen in Figure 3b that PPE−FeNPC−900 exhibited a distinct microporous (<2 nm) structure, with the most prominent peak near the peak at ~0.56 nm, where ultra-micropores (<0.7 nm) were considered to have the most efficient pore size for ion adsorption and could be used to better match the size of the dissolved ions [40,41]. This layered porous structure, composed of micro/mesopores, contributed to the enhancement of capacitive performance and ORR performance.

The Raman spectra displayed two characteristic peaks at 1340 and 1605 cm^−1^, representing the D (defects) and G peaks (graphite structure), respectively [42]. The intensity ratio of the D peak to the G peak (I_D_/I_G_) could be compared across all samples to assess the extent of defects [41]. PPE−FeNPC−900 exhibited the highest I_D_/I_G_ value of 1.01 (Figure 3c and Figure S12), indicating the highest degree of defects. This finding further suggests that enzymatic treatment and the formation of Fe−N_x_ structures increased the number of defects, corroborating the previous BET results that PPE−FeNPC−900 had the highest SSA.

### 3.3. Electrochemical Performance of Supercapacitors

**Figure 4:**
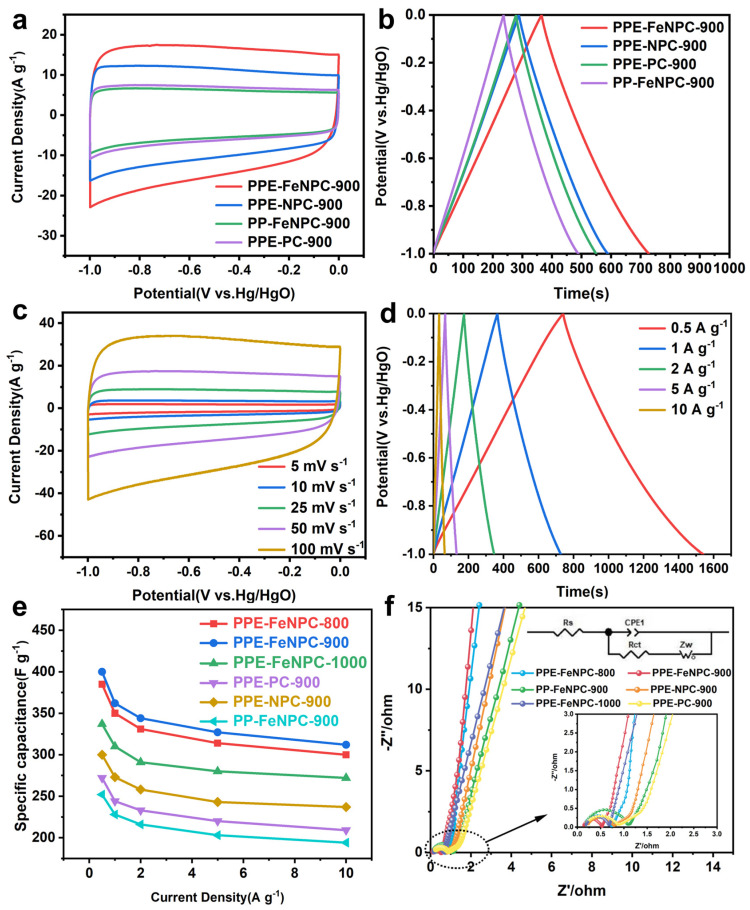
(**a**) CV curves at 50 mV s^−1^ for PPE−FeNPC−900, PPE−PC−900, PPE−NPC−900, and PP−FeNPC−900; (**b**) CV curves of PPE−FeNPC−900 at different scan rates; (**c**) GCD curves at 1 A g^−1^ for PPE−FeNPC−900, PPE−PC−900, PPE−NPC−900, and PP−FeNPC−900; (**d**) GCD curves of PPE−FeNPC−900 at different current densities; (**e**) specific capacitance of PPE−FeNPC−T (T = 800, 900, and 1000 ℃) at different current densities; (**f**) the Nyquist plots of all samples.

All samples were tested for their electrochemical properties in a 6 M KOH electrolyte solution using a three−electrode system. The CV curves (Figure 4a and Figure S13) of all the tested samples demonstrated a nearly rectangular shape and exhibited typical electric double−layer capacitance (EDLC) characteristics [43]. PPE−FeNPC−900 had the largest area, indicating its superior charge storage capacity. Moreover, a weakly broad hump could be observed on the CV curves of the PPE−FeNPC−T series samples, which suggested the presence of pseudocapacitance associated with the Fe−N_x_ structure in PPE−FeNPC−900 and the additional pseudocapacitance generated by redox reactions involving N and O heteroatoms [44]. The CV curve of PPE−FeNPC−900 at 5–100 mV s^−1^ (Figure 4b) revealed that no significant distortion or deformation occurred when the sweep frequency increased to 100 mV s^−1^, indicating outstanding rate performance and stability. The GCD (displayed in Figure 4c and Figure S14) of all the electrode samples showed highly symmetric isosceles triangles, indicative of typical EDLC characteristics, with PPE−FeNPC−900 exhibiting a specific capacitance of 362 F g^−1^ calculated using Equation (S1), which is consistent with the maximum curve area of its CV curve and demonstrates its reliability regarding maximum capacitance. The GCD curves of PPE−FeNPC−900 at current densities of 0.5–10 A g^−1^ are shown in Figure 4d, displaying highly symmetric isosceles triangles and illustrating excellent electrochemical reversibility. The specific capacitance reached 400 F g^−1^ at a current density of 0.5 A g^−1^. During rapid charging and discharging, the electrolyte was unable to completely permeate the electrode surface at elevated current densities, leading to a reduction in specific capacitance [40]. However, when the current density reached 10 A g^−1^, a high specific capacitance of 312 F g^−1^ (78% capacitance retention) could still be achieved, displaying an excellent rate performance, even surpassing that of the biomass−derived carbon materials described in recent publications (Table S3) [45,46,47,48,49]. Figure 4e presents the calculated capacitance values of the electrodes prepared from all the samples under different current densities. The excellent capacitive performance of PPE−FeNPC−900 was not only attributed to its high SSA and micropore−rich mesoporous structure but also to the positive influence of the Fe−N_x_ structure on capacitive performance.

The ion transport and charge transfer rates of the samples were studied using EIS. Figure 4f displays the Nyquist plots recorded for all the samples at an open−circuit voltage. The diameter of the approximate semicircle portion of the plot represents the charge transfer resistance (R_ct_), and the cross−axis intercept is related to the equivalent series resistance (R_s_) [50]. A larger slope of the linear part indicates lower resistance to ion diffusion. PPE−FeNPC−900 exhibited a steep linear shape, indicative of near−ideal capacitive performance. Moreover, PPE−FeNPC−900 had the smallest R_s_, indicating its lowest internal and interfacial resistance, with higher accessibility of the surface to electrolyte ions [51]. Utilizing the equivalent circuit model depicted in the inset of Figure 4f, the Nyquist curve was fitted via ZView2 software. Comprehensive fitted data for R_s_, R_ct_, and Z_w_ (ion diffusion resistance) are provided in Table S4. The results revealed that PPE−FeNPC−900 demonstrated the lowest R_ct_ value. Since R_ct_ was closely associated with the charge transfer capacity of the electrodes, the lowest R_ct_ indicated that electrodes prepared from PPE−FeNPC−900 displayed an enhanced charge transfer and superior electrical conductivity [52]. The low resistance of PPE−FeNPC−900 was not only attributed to its higher graphitization but also to its abundant microporous mesopores, which provided transport channels that shortened ion dispersion paths and reduced charge transfer resistance.

**Figure 5:**
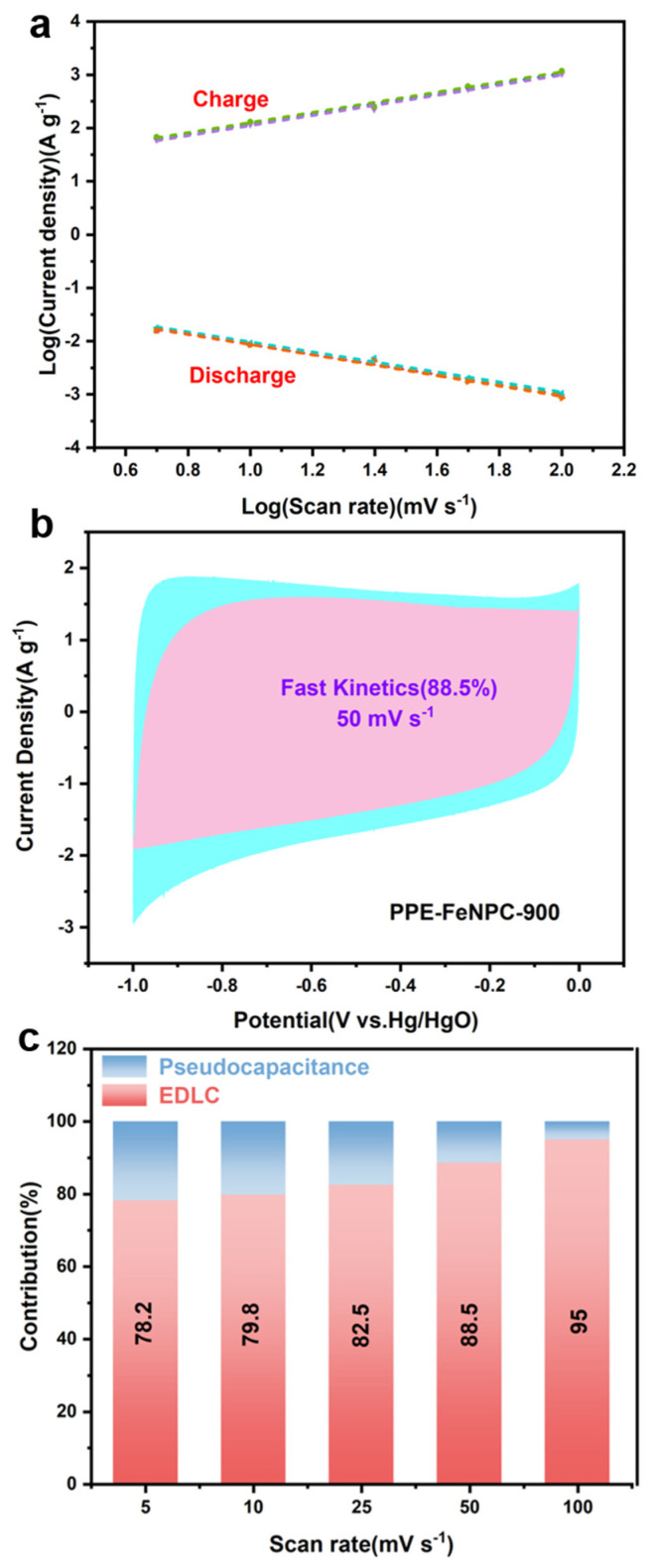
(**a**) The linear relationship between current density and scan rate of PPE−FeNPC−900 at different potentials during charging and discharging; (**b**) capacitance contribution at 50 mV s^−1^ and (**c**) capacitance contribution at different scan rates of PPE−FeNPC−900.

The fast charging/discharging kinetics of PPE−FeNPC−900 were investigated using Equations (S2) and (S3). Figure 5a displays the linear relationship between log (i) and log (v) during the charging/discharging process after a perfect linear fit (R^2^ > 0.99) at different potentials. Generally, b−values were utilized to assess the kinetics of redox reactions, and a b−value approaching 1 indicated that the reaction kinetics corresponded to a fast surface−controlled process [53]. The b−value fluctuated in the range of 0.94–0.98 with voltage (Figure 5a), confirming that this process was dominated by a surface−controlled mechanism exhibiting ultra−fast reaction kinetics.

Moreover, the contributions of different capacitors were further compared quantitatively using Equation (S4). As shown in Figure 5b, the contribution of PPE−FeNPC−900’s surface−controlled capacitance reached 88.5% at a sweep rate of 50 mV s^−1^. Figure 5c demonstrates that the surface control capacitance contribution gradually increased with the increase in the sweep rate, reaching 95% when the sweep rate reached 100 mV s^−1^. The significant improvement in rate capability could be attributed to the significant contribution of surface−controlled capacitance. This was mainly due to the highly accessible surface area and appropriate pore structure of PPE−FeNPC−900, which resulted in a substantial EDLC. The surfaces containing abundant micropores and mesopores acted as an ideal electrolyte ion storage buffer, which facilitated short spreading distances of ions to the inner surface, thereby enhancing electron transfer.

**Figure 6:**
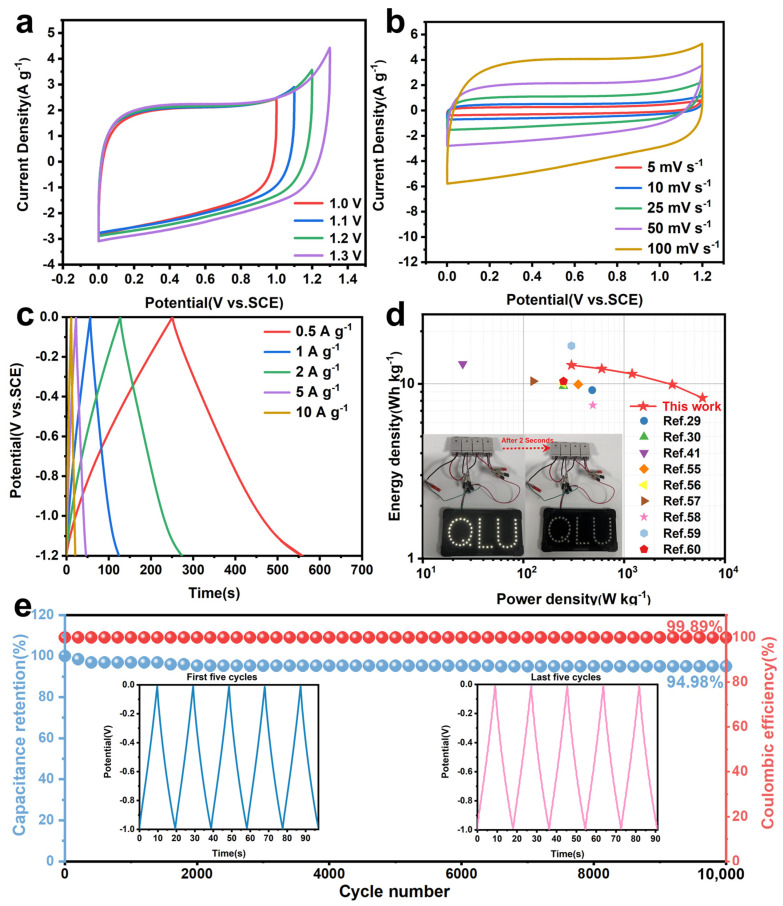
(**a**) CV curves of PPE−FeNPC−900−SC in different voltage windows at a scan rate of 50 mV s^−1^; (**b**) CV curves of PPE−FeNPC−900−SC at different scan rates; (**c**) GCD curves of PPE−FeNPC−900−SC at different current densities; (**d**) Ragone plots of FeNPC−900−SC (the inset shows that four PPE−FeNPC−900−SC in series instantaneously lit 3 V LED lights); (**e**) stability of PPE−FeNPC−900−SC (the inset shows a comparison of the GCD curves at the beginning and end of the period of cycle stability).

Symmetrical supercapacitors, PPE−FeNPC−900−SC, were assembled using PPE−FeNPC−900 as both positive and negative materials and 6 M KOH as the electrolyte. Their performance as supercapacitors was also investigated. The CV curves were acquired at various potential windows (Figure 6a). The results demonstrate that when the working voltage window increased to 1.2 V, the CV curves maintained a quasi−rectangular shape. However, when the working voltage window rose to 1.3 V, the CV curves shifted significantly due to hydrogen and oxygen reactions taking place at the electrodes of PPE−FeNPC−900−SC, establishing the upper limit potential of PPE−FeNPC−900−SC at 1.2 V [54]. The CV curves at different scan rates (Figure 6b) show that a quasi−rectangular shape was retained even at a scan rate of 100 mV s^−1^, indicating good EDLC characteristics and rate performance. The GCD curves (Figure 6c) exhibited approximate isosceles triangle shapes at various current densities, demonstrating excellent chemical reversibility and ideal capacitance. Using Equation (S5), the specific capacitance of PPE−FeNPC−900−SC was calculated to be 63 F g^−1^ at 0.5 A g^−1^, which was maintained at a high value of 40.5 F g^−1^ even at a current density of 10 A g^−1^. Based on the calculated GCD results, the energy density and power density of PPE−FeNPC−900−SC were further calculated via Equations (S6) and (S7) to assess their performance for practical applications. Figure 6d shows that the energy density of PPE−FeNPC−900−SC reached 12.8 Wh kg^−1^ at a power density of 300 W kg^−1^ and remained at 8.3 Wh kg^−1^ at a power density of 6000 W kg^−1^. As depicted in Figure 6d and Table S5, under similar test conditions, PPE−FeNPC−900−SC exhibited significant advantages over other reported biomass−derived carbon−based SCs [29,30,41,55,56,57,58,59,60]. The cycling stability test of PPE−FeNPC−900−SC, performed at a current density of 10 A g^−1^, yielded a capacitance retention rate of 96% and a coulombic efficiency of approximately 100% after 10,000 cycles, indicating outstanding electrochemical stability and high reversibility (Figure 6e).

### 3.4. Electrocatalytic Performance for Oxygen Reduction Reaction

**Figure 7:**
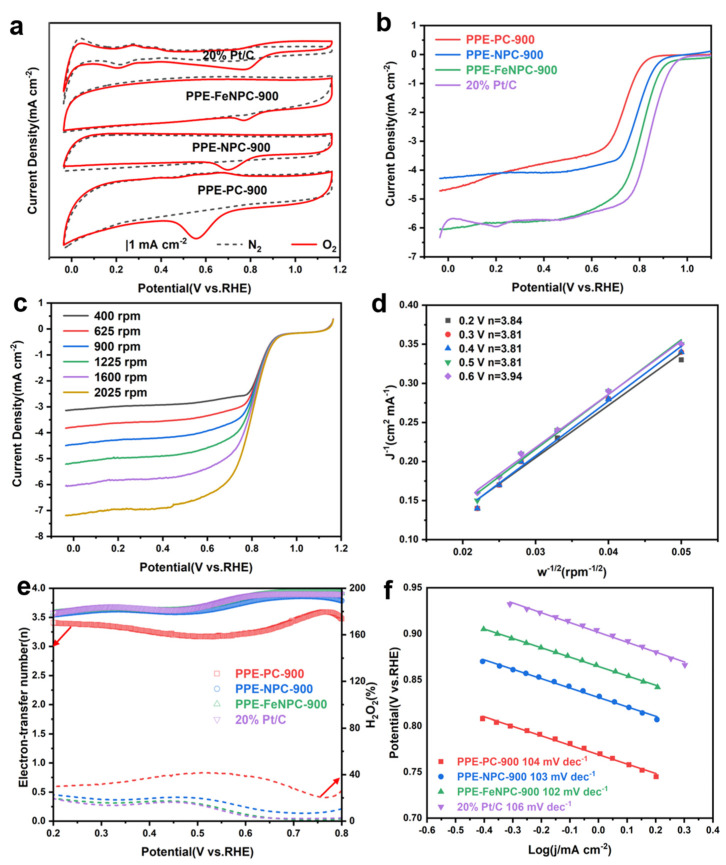
(**a**) CV spectra in aqueous solution of 0.1 M KOH saturated with N_2_ and O_2_; (**b**) RRDE tests were performed in O_2_−saturated electrolyte at 1600 rpm; (**c**) the polarization curves at different rotating rates and (**d**) K−L plots for the PPE−FeNPC−900; (**e**) transferred electron numbers and hydrogen peroxide content for PPE−PC−900, PPE−NPC−900, PPE−FeNPC−900, and Pt/C; (**f**) Tafel slopes of PPE−PC−900, PPE−NPC−900, PPE−FeNPC−900, and Pt/C samples.

The performance of PPE−FeNPC−900 as a catalyst in an ORR was assessed using cyclic voltammetry (Figure 7a and Figure S15a). All the pomelo−peel−derived samples exhibited bilayer capacitance curves without distinct features in the N_2_−saturated electrolyte, but in the O_2_−saturated electrolyte, a pronounced cathodic peak could be observed, indicating the samples had catalytic activity for the ORR. The peak oxygen reduction for sample PPE−FeNPC−900 occurred at 0.78 V vs. RHE, which was comparable to the peak ORR of 20% Pt/C. The RRDE test provided additional information on the ORR activity of our porous carbon (Figure 7b and Figure S15b). The onset potential (Eonset) of sample PPE−FeNPC−900 was 0.982 V, second only to 20% Pt/C (with a value of 0.998 V) and higher than that of other samples. Under the same test conditions, the half−wave potential of PPE−FeNPC−900 (E_1/2_, 0.85 V) was equal to that of 20% Pt/C and higher than that of other samples. At +0.300 V, the diffusion limit current of sample PPE−FeNPC−900 exceeded that of commercial Pt/C (20 wt%). Table S6 showcases the superior ORR performance of PPE−FeNPC−900 in comparison to other previously documented electrocatalysts [56,57,58,59,60,61,62,63,64,65,66]. Figure 7c presents the LSV curves of the PPE−FeNPC−900 samples obtained at different rotational speeds, and the corresponding Koutecky−Levich (K−L) plots (Figure 7d) based on Equations (S8) and (S9) exhibit linear fits in the range of 0.2–0.5 V. PPE−FeNPC−900 demonstrated an excellent linear relationship, indicating that the reduction rate of O_2_ is proportional to the dissolved concentration of O_2_ in the electrolyte, and it also revealed that the catalyst’s action had first−order reaction kinetics. The catalysis conformed to first−order reaction kinetics. The electron transfer number of sample PPE−FeNPC−900 at 0.6 V was 3.94, which confirmed that the reaction was a four−electron transfer process.

To analyze the ORR kinetics, the electron transfer number (n) and H_2_O_2_ yields were determined via the calculation of the ring current and disk current data via Equations (S10) and (S11) (Figure 7e and Figure S15c). The electron transfer number of sample PPE−FeNPC−900 approached 4, which was higher than that of the other samples in the range of 0.2–0.8 V, confirming that the reaction was a four−electron transfer process. The electron transfer number of sample PPE−FeNPC−900 at 0.7 V was 3.91, exceeding that of commercial Pt/C (3.89) and other samples. The H_2_O_2_ yields of less than 18% obtained using PPE−FeNPC−900−based catalysts in the range of 0.2–0.8 V were comparable to those of commercial Pt/C and lower than those of the other samples, indicating that the material exhibited excellent selectivity for H_2_O during the ORR. The Tafel slope of PPE−FeNPC−900 sample was 102 mV dec^−1^ (Figure 7f and Figure S15d), which is lower than the commercial Pt/C value (106 mV dec^−1^) and the slopes of other samples. This suggests that PPE−FeNPC−900 and Pt/C share similar catalytic mechanisms, and the initial electron reduction process of oxygen was likely the rate−determining step. Therefore, the PPE−FeNPC−900 catalyst demonstrated superior ORR performance, even surpassing that of Pt/C, due to its high specific surface area and abundant microporous mesopore structure, which enabled the full exposure of the uniformly dispersed Fe−N_x_ structure and electrocatalytic active sites.

**Figure 8:**
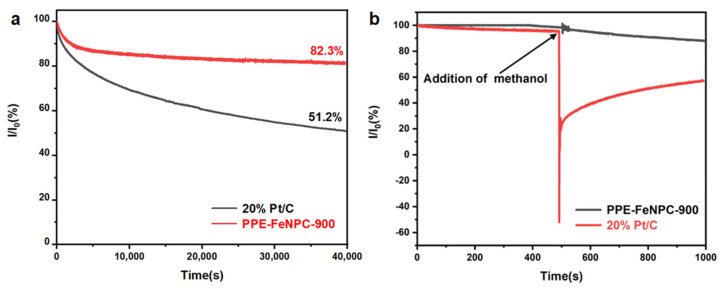
(**a**) I-t chronoamperometric curves of PPE-FeNPC-900 and Pt/C in O_2_-saturated 0.1 M KOH solution with 900 rpm rotation speed. (**b**) At 900 rpm, 1 M methanol was added for PPE−FeNPC−900 and Pt/C (20 wt%) before and after developing the chrono−current response plots.

To assess the potential of sample PPE−FeNPC−900 as a catalyst for practical applications, it was essential to demonstrate its stability during operation. Therefore, chronoamperometric tests were conducted in O_2_−saturated 0.1 M KOH. After 40,000 s, the current of the electrode loaded with commercial Pt/C diminished to only 51.2% of its initial current (Figure 8a). In contrast, the electrode loaded with the PPE−FeNPC−900 catalyst retained 82.3% of its initial current density, indicating that the PPE−FeNPC−900 material possessed superior operational stability compared to commercial Pt/C. Fuel cross−tolerance is another prerequisite for fuel cells to be viable in large−scale practical applications. When 1 M of methanol was introduced into the electrolyte as an example fuel, the voltametric current of the electrode loaded with Pt/C exhibited a significant decline (Figure 8b). However, for the electrode loaded with PPE−FeNPC−900, the presence of methanol did not cause any noticeable attenuation of the ORR current. These results suggest that the PPE−FeNPC−900-based electrode could be employed with other fuels.

## 4. Conclusions

In the current work, high−specific surface area Fe, N−doped porous carbon materials were successfully synthesized using the versatile pomelo peel as the carbon source via enzymatic treatment. This study not only eliminated the traditional treatment with strong corrosive chemicals and made it easier to scale up but also expanded the application scope, as the prepared porous carbon materials could be optimally applied to supercapacitors and ORR catalysts. The prepared PPE−FeNPC−900, with its abundant micro− and mesopores, high specific surface area, multi−atom doping, and homogeneously dispersed Fe−N_x_ structure, served as an excellent supercapacitor electrode (for which high specific capacitance, low resistance, high energy density, and exceptional cycling stability of the assembled supercapacitor was observed) and ORR catalyst (exhibiting a high onset/half−wave potential, increased electron transfer numbers, reduced hydrogen peroxide yields, and significantly improved operational stability and methanol tolerance compared to commercial Pt/C). Importantly, the abundant raw materials used in and simplicity of the preparation process provided a novel, green, straightforward, and efficient method for the large−scale production of high−performance energy materials, which is expected to offer new opportunities for the future study of multifunctional carbon materials derived from biomass and open new possibilities for the development of environmentally friendly materials for multifunctional applications.

## Data Availability

Not applicable.

## Supplementary Materials

The following supporting information can be downloaded at https://www.mdpi.com/article/10.3390/polym15193879/s1. Figure S1: FT-IR spectra of PPC and PPC-E150-18; Figure S2: XRD patterns of PPC-E after low-temperature pyrolysis; Figure S3: SEM images of PPC and PPC-Ex-y (x = 75 and 150; y = 12, 18, and 24); Figure S4: N_2_ adsorption and desorption curves for PPC and PPC-Ex-y (x = 75 and 150; y = 12, 18, and 24); BJH pore size distributions for PPC and PPC-Ex-y (x = 75 and 150; y = 12, 18, and 24); Figure S5: The pomelo peels were treated at 150 μL enzymatic treatment for different times; Figure S6: XRD patterns of PPE-FeNPC-T (T = 800, 900 and 1000 °C) before acid washing; PPE-FeNPC-T (T = 800, 900, and 1000 °C) after acid washing; Figure S7: XPS C 1s spectra of PPE-FeNPC-800, PPE-FeNPC-1000, PPE-PC-900, and PPE-NPC-900; Figure S8: XPS O 1s spectra of PPE-FeNPC-800, PPE-FeNPC-1000, PPE-PC-900, and PPE-NPC-900; Figure S9: XPS Fe 2p spectra of PPE-FeNPC-800, PPE-FeNPC-900, PPE-FeNPC-1000, and PP-FeNPC-900; Figure S10: XPS N 1s spectra of PPE-FeNPC-800, PPE-FeNPC-1000, PPE-PC-900, and PP-NPC-900; Figure S11: N_2_ adsorption and desorption curves of PPE-FeNPC-T (T = 800, 900, and 1000 °C); HK-method- (pore width < 2 nm) and BJH-method (pore width ≥ 2 nm) -derived pore size distributions of PPE-FeNPC-T (T = 800, 900, and 1000 °C); Figure S12: Raman spectra of PPE-FeNPC-T (T = 800, 900, and 1000 °C); Figure S13: CV curves at 50 mV s^−1^ for PPE-FeNPC-T (T = 800, 900, and 1000 °C); Figure S14: GCD curves at 1 A g^−1^ for PPE-FeNPC-T (T = 800, 900, and 1000 °C); Figure S15: CV obtained in N_2_- and O_2_-saturated 0.1 M KOH; RRDE voltammograms recorded in O_2_-saturated electrolyte at 1600 rpm at 10 mV s^−1^ sweep rate for the electrodes containing PP-FeNPC-900 and PPE-FeNPC-900 catalytic materials; electron transfer numbers and H_2_O_2_ yields for the catalysts based on the PP-FeNPC-900 and PPE-FeNPC-900; Tafel slopes of PP-FeNPC-900 and PPE-FeNPC-900samples; Table S1: Surface composition and the content of different nitrogen doping configurations of samples; Table S2: SSA and pore parameters of all samples; Table S3: Comparison of performance of carbon materials for SCs; Table S4: R_s_ (Ω), R_ct_ (Ω), and Z_w_ (Ω) of all samples; Table S5: Summary of capacitive performance of the reported carbon-based material; Table S6: Comparison of the ORR performance of catalysts.
