# Supplementary material for: Pyrolysis Enzymolysis-Treated Pomelo Peel: Porous Carbon Materials with Fe−Nx Sites for High-Performance Supercapacitor and Efficient Oxygen Reduction Applications
Source: Polymers (Basel). 2023 Sep 25;15(19):3879. doi: 10.3390/polym15193879 (PMC10575101; doi:10.3390/polym15193879)
Supplement: Supplementary file 1 [file polymers-15-03879-s001.zip › polymers-2615592-supplementary.pdf]

Article

Pyrolysis Enzymolysis-Treated Pomelo Peel: Porous Carbon Materials with Fe−N_x Sites for High-Performance Supercapacitor and Efficient Oxygen Reduction Applications

Xiangyu Chen, Jiahua Ma, Xiaoshuai Sun, Chuanshan Zhao *, Jiehua Li and Hui Li

State Key Laboratory of Biobased Material and Green Papermaking, Qilu University of Technology, Shandong Academy of Sciences, Jinan 250353, China; colacxy@163.com (X.C.); 13290394238@163.com (J.M.); 17861407148@163.com (X.S.); 13695310529@163.com (J.L.); 15668434088@163.com (H.L.)

* Correspondence: ppzcs78@163.com

1. Material Characterization:

Transmission electron (JEM-2100, 200 kV) and scanning electron (Zeiss EVO-18, 10 kV) microscopes (TEM and SEM, respectively) were used to observe microstructures. SSA and pore size distribution data were collected using a Kubo X1000 device and processed using Brunauer–Emmett–Teller (BET), Horvath–Kawazoe (HK), and Barrett–Joyner–Halenda (BJH) calculation methods. X-ray powder diffraction (XRD) measurements were carried out at room temperature using a Bruker D8 Advance X-ray powder diffractometer with Ni-filtered Cu-Ka radiation (λ=0.154 nm) from 5º-80º. The elemental composition of the samples was determined via X-ray photoelectron spectroscopy (XPS, Thermo Fisher Scientific). Raman spectra were obtained using a micro-Raman setup with a LabRAM Aramis microspectrograph at a wavelength of 532 nm (2.34 eV). The determination of the functional group species of carbon materials was carried out via Fourier transform infrared (FT-IR) spectroscopy. The tests were performed using the KBr press method in the range of 400-4000 cm^{-1}.

2. Electrochemical measurement for Supercapacitors

The electrodes were made as follows: The activated carbon was mixed with acetylene black and polytetrafluoroethylene (PTFE) in ethanol in a mass ratio of 8:1:1 and stirred thoroughly until the ethanol evaporated completely. The mixture was placed on a thermostatic heating plate at 60°C to form a carbon film and dried under a vacuum at 80 °C for 12 h. The carbon film (1×1 cm^2) was pressed on a nickel foam collector (1×2 cm^2) for 30 s. The mass loading of active material for each working electrode was 2.5 mg cm^{-2}. To evaluate the potential of the prepared electrode materials for practical applications, a two-electrode

system was used for testing. A symmetric supercapacitor (CR2032 stainless steel coin cell) was assembled from two working electrodes of the same mass and a cellulose diaphragm (NKK TF40330, Japan).

All measurements were performed in 6 M KOH electrolyte solution. Cyclic voltammetry (CV), galvanostatic charge–discharge (GCD), and electrochemical impedance spectroscopy (EIS) were carried out. In the three-electrode system, platinum foil and Hg/HgO electrodes were used as the counter and reference electrodes.

The mass ratio capacitance (C, F g^{-1}) in the three-electrode system was calculated from the charge/discharge curve using equation (S1)

$$C = I \triangle t/(m \triangle V) \qquad \text{(S1)}$$

where I (A) is the current during charging and discharging, △t(s) is the discharge time, m (g) is the weight of the active material in the electrode, and △V (V) is the charging and discharging potential window without a voltage drop.

The charge storage mechanism of the supercapacitor was evaluated using the following equation：

$$i = kv^b \qquad \text{(S2)}$$

$$log(i) = b\,log(v) + log(k)$$

(S3)

In the equation above, the response current i（A g^{-1}） measured at a fixed voltage obeys a power-law relationship with the scan rate v（mV s^{-1}）, and k and b are constants.

The contribution value of capacitance (i.e., surface-controlled capacitance and diffusion-controlled capacitance) was evaluated using the following equation：

$$i = k_1 v + k_2 v^{1/2} \qquad \text{(S4)}$$

Above, $k_1 v$ and $k_2 v^{1/2}$ correspond to the capacitive behavior contributed by the fast kinetic process and the diffusive behavior contributed by the slow kinetic process, respectively. The diffusive behavior stems from the internal pseudocapacitance that accompanies the Faraday charge transfer process, and k_1 and k_2 are constants.

The specific capacitance (Cs, F g^{-1}) of the assembled device was calculated using the following equation(S5):

$$Cs = I \triangle t/(m \triangle V) \qquad \text{(S5)}$$

Above, I (A) is the current during charging and discharging, △t(s) is the discharge time,

m (g) is the total weight of the active material in both electrodes, and △V (V) is the charging and discharging potential window without a voltage drop.

The energy density (E, Wh kg^{-1}) and power density (P, W kg^{-1}) of a symmetric supercapacitor were calculated using equations (S6) to (S7):

$$E = Cs \times (\triangle V)^2/(2 \times 3.6) \quad \text{(S6)}$$

$$P = E/\triangle t \times 3600 \quad \text{(S7)}$$

3. Electrochemical tests for Oxygen Reduction Reaction

For ORR performance measurements, Pt foil and Ag/AgCl were selected as counter and reference electrodes. For the working electrode, 5 mg of the prepared sample was ultrasonically dispersed in 1.0 ml of 1.0 vol.% Nafion ethanol solution added to form a homogeneous ink. Then, 10 μL of the above ink was dropped onto a glassy carbon electrode (5 mm diameter) and dried at room temperature. CV measurements were performed at 50 mV s^{-1} in N_2- or O_2-saturated 0.1 M KOH, and linear scanning voltammograms (LSV) were performed on a rotating disc electrode and a rotating ring-disc electrode (RDE and RRDE, respectively) in O_2-saturated 0.1 M KOH at 400 to 2025 rpm and a sweep speed of 10 mV s^{-1}. The long-term stability was determined at a constant voltage of -0.4 V vs. Ag/AgCl in an O_2-saturated 0.1 M KOH electrolyte at a rotation speed of 900 rpm. During the crossover tests, 1.0 M of methanol was added into the O_2-saturated 0.1 M KOH after 500 s.

The slopes of the Koutecky–Levich curves were analyzed at different electrode potentials and fitted linearly to calculate the number of electrons transferred (n)

$$\frac{1}{j} = \frac{1}{j_k} + \frac{1}{B\omega^{1/2}} \quad \text{(S8)}$$

where j、j_k denote measured current and dynamic limit current, respectively, and ω denotes electrode rotation speed. The lavish slope (B) was evaluated using equation (S9).

$$B = 0.62nFC_{O_2}D_{O_2}^{2/3}v^{-1/6} \quad \text{(S9)}$$

In the equation above, n is the number of electron transfers during ORR, F is Faraday's constant (96485 C mol^{-1}), C_{O2} is the oxygen concentration (solution) in 0.1 M KOH, D_{O2} is the oxygen diffusion coefficient in 0.1 M KOH, and v is the kinematic viscosity of 0.1 M KOH (1.2×10^{-3}mol cm^{-3}, $1.9 \times 10^{-5}$$cm^2$ s^{-1} and 0.01cm^2 s^{-2}).

The four-electron selectivity of the catalyst was evaluated based on the electron transfer

number (n) and the H_2O_2 yield, as shown in the following equation:

$$\%(H_2O_2) = 200 \times \frac{I_R/N}{I_D+I_R/N} \quad \text{(S10)}$$

$$n = 4 \times \frac{I_D}{I_D+I_R/N}$$

(S11)

I_D is the disk current, I_R is the ring current, and N=0.37 is the current collection efficiency of the platinum ring.

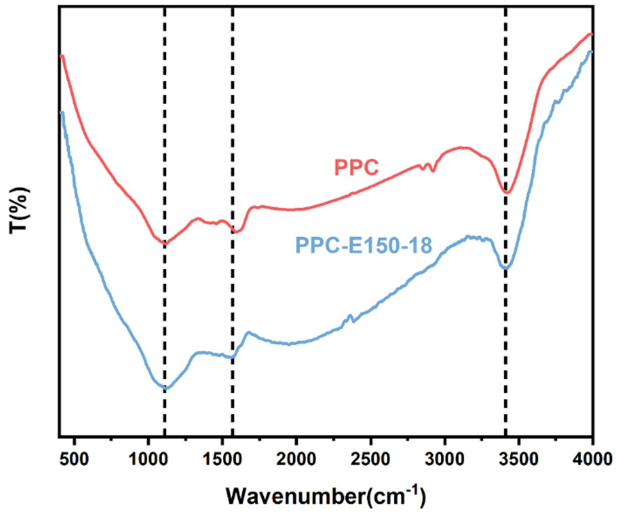

Figure S1. FT-IR spectra of PPC and PPC-E150-18.

Protopectinase hydrolyzes insoluble protopectin to water-soluble pectin, breaking the chemical bond between polymethoxygalacturonic acid and arabinose. In contrast, polygalacturonase breaks the α-1,4 glycosidic bond of pectic acid and promotes the hydrolysis of polygalacturonase chains. According to the mechanism of hydrolysis, there are two types: polygalacturonase exonuclease and polygalacturonate endonuclease. Endonuclease hydrolyzes the α-1,4 bond irregularly from the inside of the molecule, causing a rapid decrease in the viscosity of pectin or pectic acid; exonuclease hydrolyzes the α-1,4 bond from the end of the molecule one by one, generating galacturonic acid. The lyase cleaves the pectin polymer via trans-elimination. The lyase breaks the glycosidic bond at the C-4 position and simultaneously eliminates an H atom from C-5 to produce an unsaturated product. Pectin esterase randomly excises the ester bond between the methoxy and galacturonic acid in the water-soluble pectin molecule, producing methanol and free carboxyl groups.

The FT-IR spectrum of the enzyme-treated carbon precursor (PPC-E150-18) is similar to

that of the non-enzyme-treated carbon precursor (PPC), as seen in the IR spectrum of Figure S1. It has a broad and strong transmission peak at 3400 cm^{-1} attributed to the O-H stretching vibration of hydroxyl functional groups, mainly corresponding to the absorption peaks of alcohols and phenols, and this is mainly due to the hydroxyl groups in cellulose and hemicellulose. It also has a peak at 1560 cm^{-1} corresponding to the stretching vibration of C=O in the free carboxyl group of unsaturated aldehydes, while the strong absorption peak near 1110 cm^{-1} is due to the deformation vibration of C-O, which corresponds to the carboxyl group. This indicates that both samples contain more hydrophilic groups (-COOH, -OH), and in comparison, the absorption peaks of the surface functional groups at 1110 cm^{-1} and 1560 cm^{-1} are broader and stronger in the treated samples, indicating that the enzyme treatment favors the formation of carboxyl groups, corroborating the notion that pectin esterase excises the ester bond between methoxy and galacturonic acid to produce methanol and free carboxyl groups.

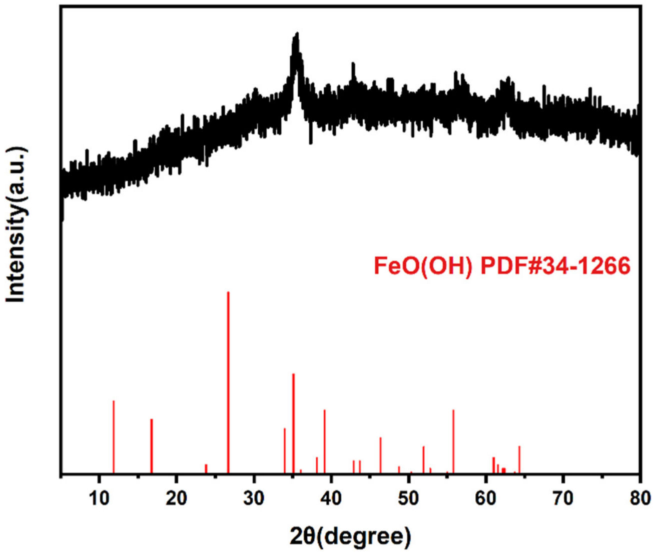

Figure S2. XRD patterns of PPC-E after low-temperature pyrolysis.

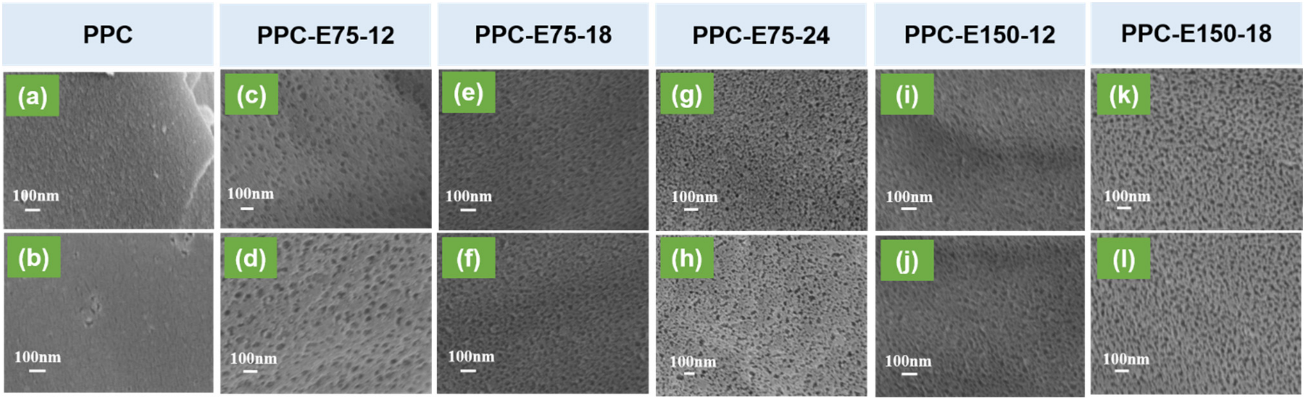

Figure S3. (a-l) SEM images of PPC and PPC-Ex-y (x = 75 and 150； y = 12, 18, and 24).

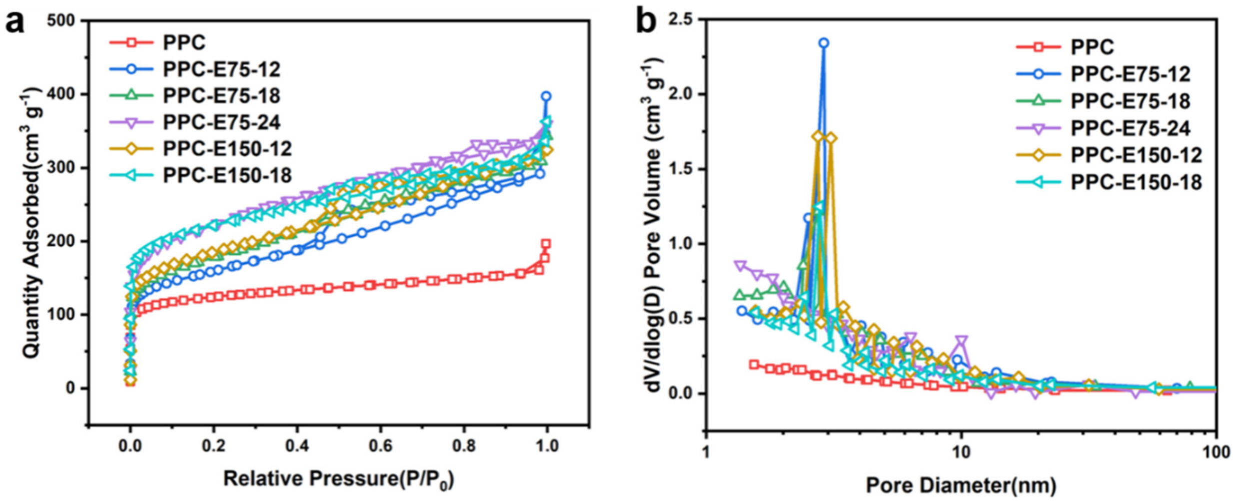

Figure S4. (a) N_2 adsorption and desorption curves for PPC and PPC-Ex-y (x = 75 and 150； y = 12, 18, and 24); (b) BJH pore size distributions for PPC and PPC-Ex-y (x = 75 and 150； y = 12, 18, and 24).

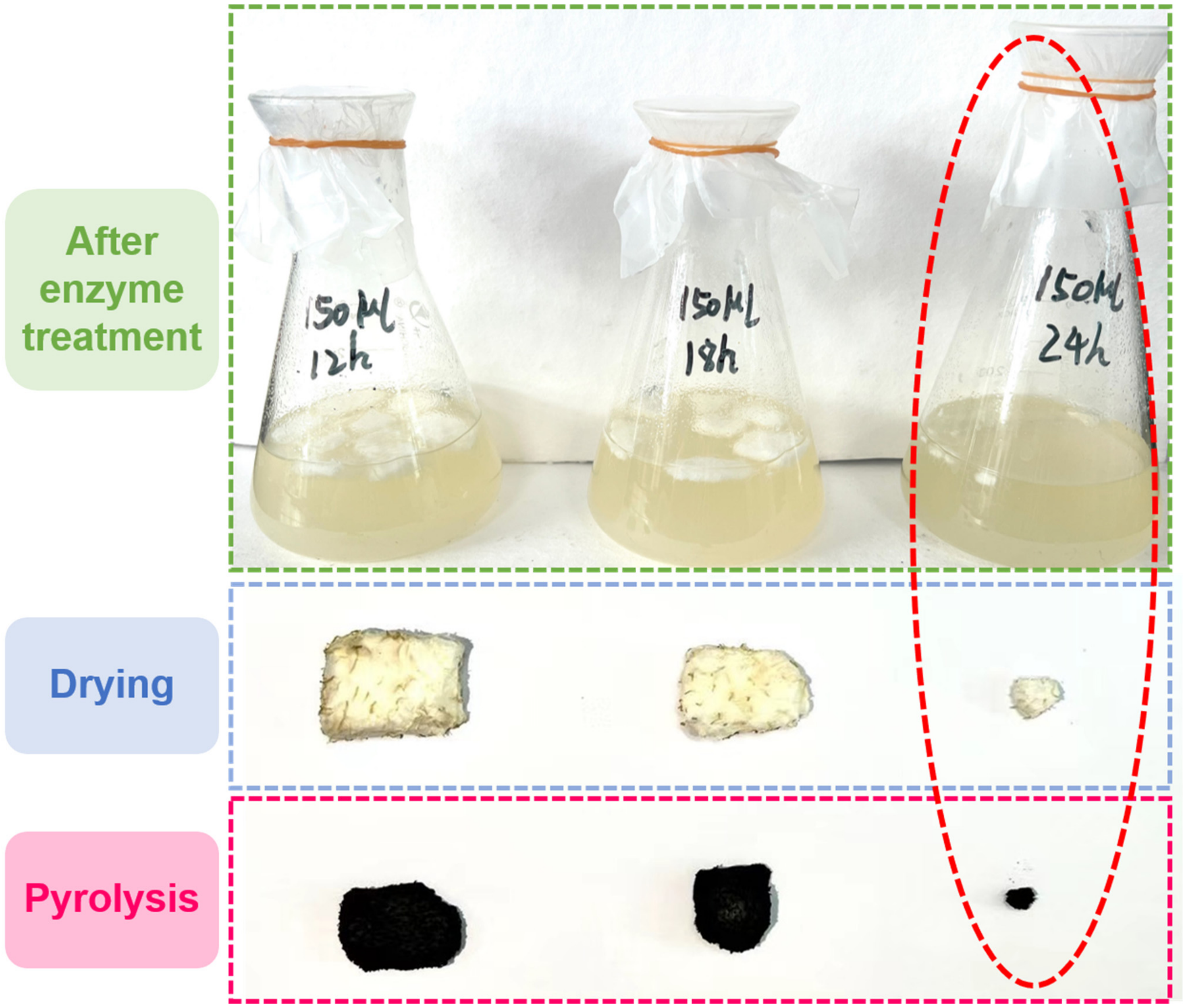

Figure S5. The pomelo peels were treated with a 150 μL enzymatic treatment for different durations.

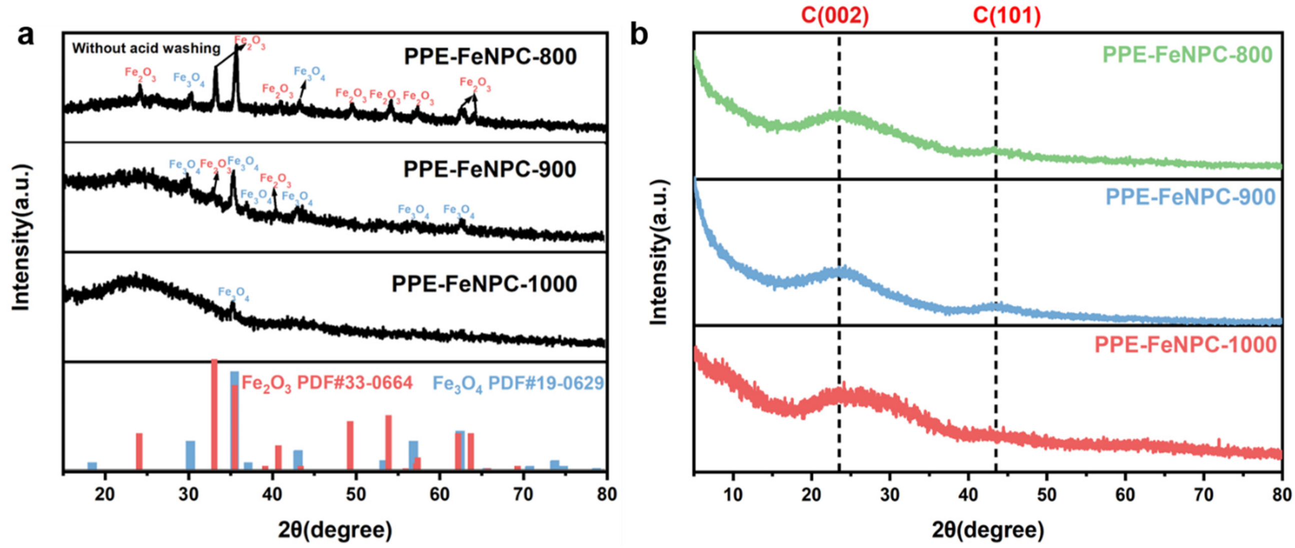

Figure S6. XRD patterns of (a) PPE-FeNPC-T (T=800, 900, and 1000ºC) before acid washing; (b) PPE-FeNPC-T (T=800, 900, and 1000ºC) after acid washing.

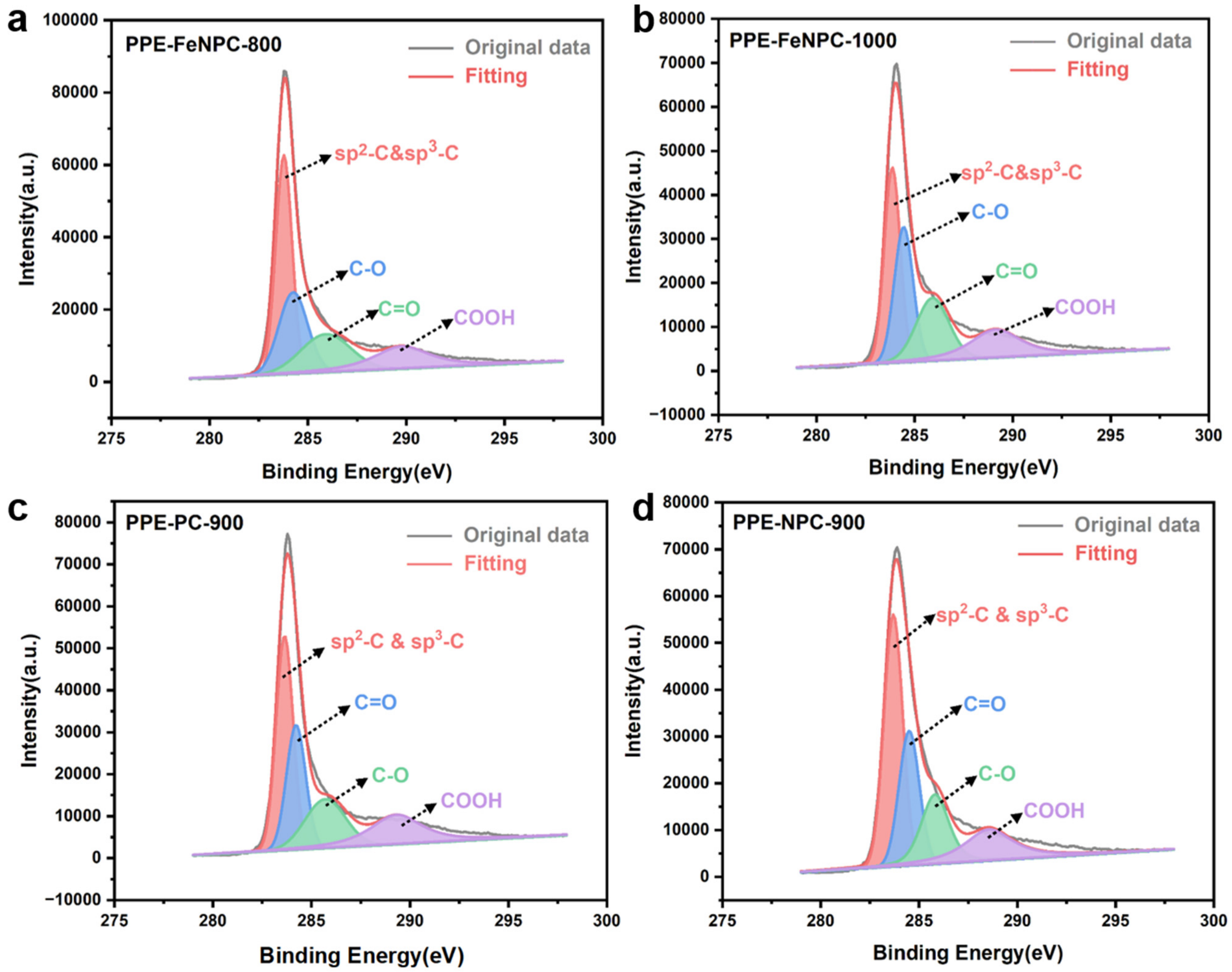

Figure S7. XPS C1s spectra of (a) PPE-FeNPC-800; (b) PPE-FeNPC-1000; (c) PPE-PC-900; and

(d) PPE-NPC-900.

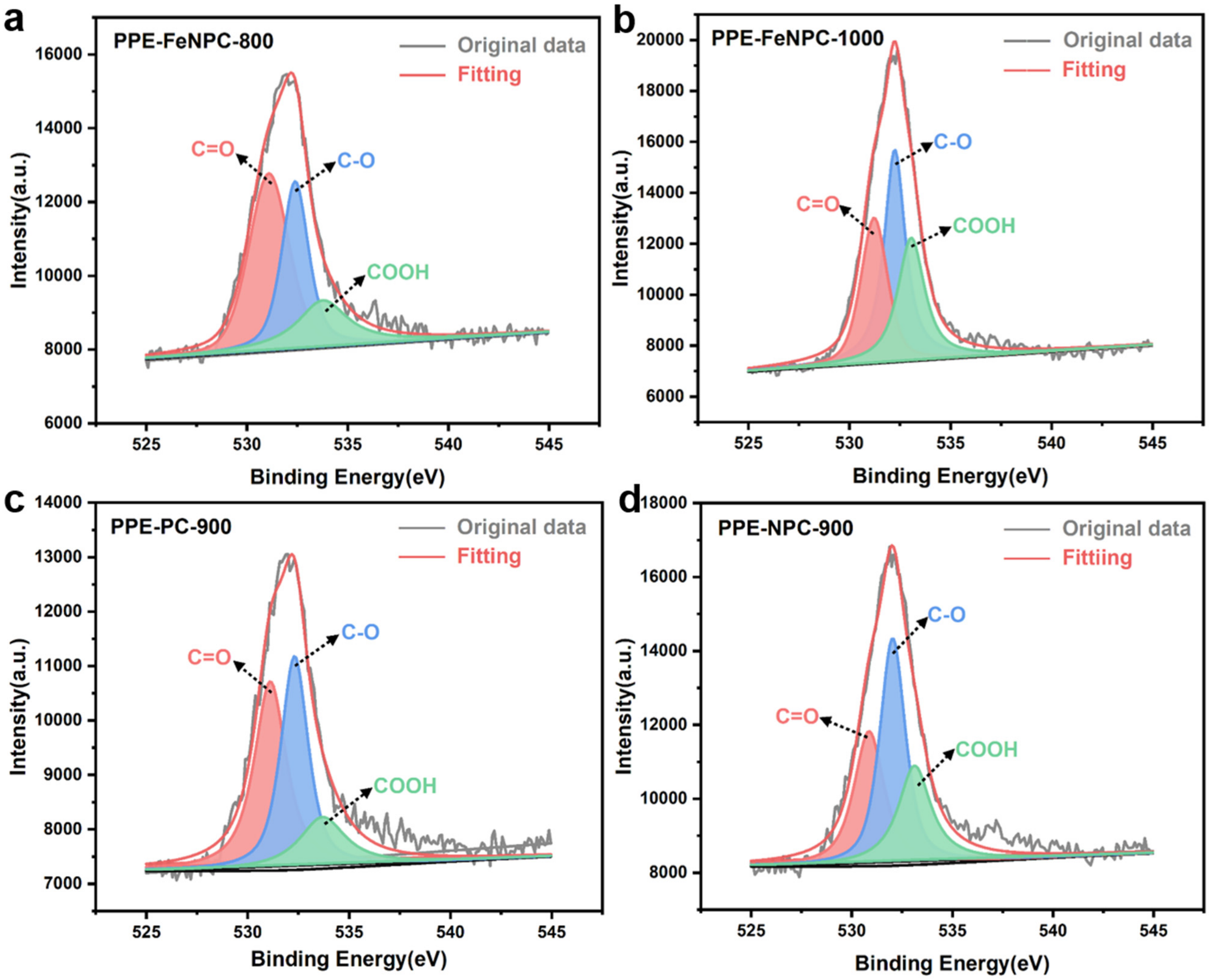

Figure S8. XPS O1s spectra of (a) PPE-FeNPC-800; (b) PPE-FeNPC-1000; (c) PPE-PC-900; and (d) PPE-NPC-900.

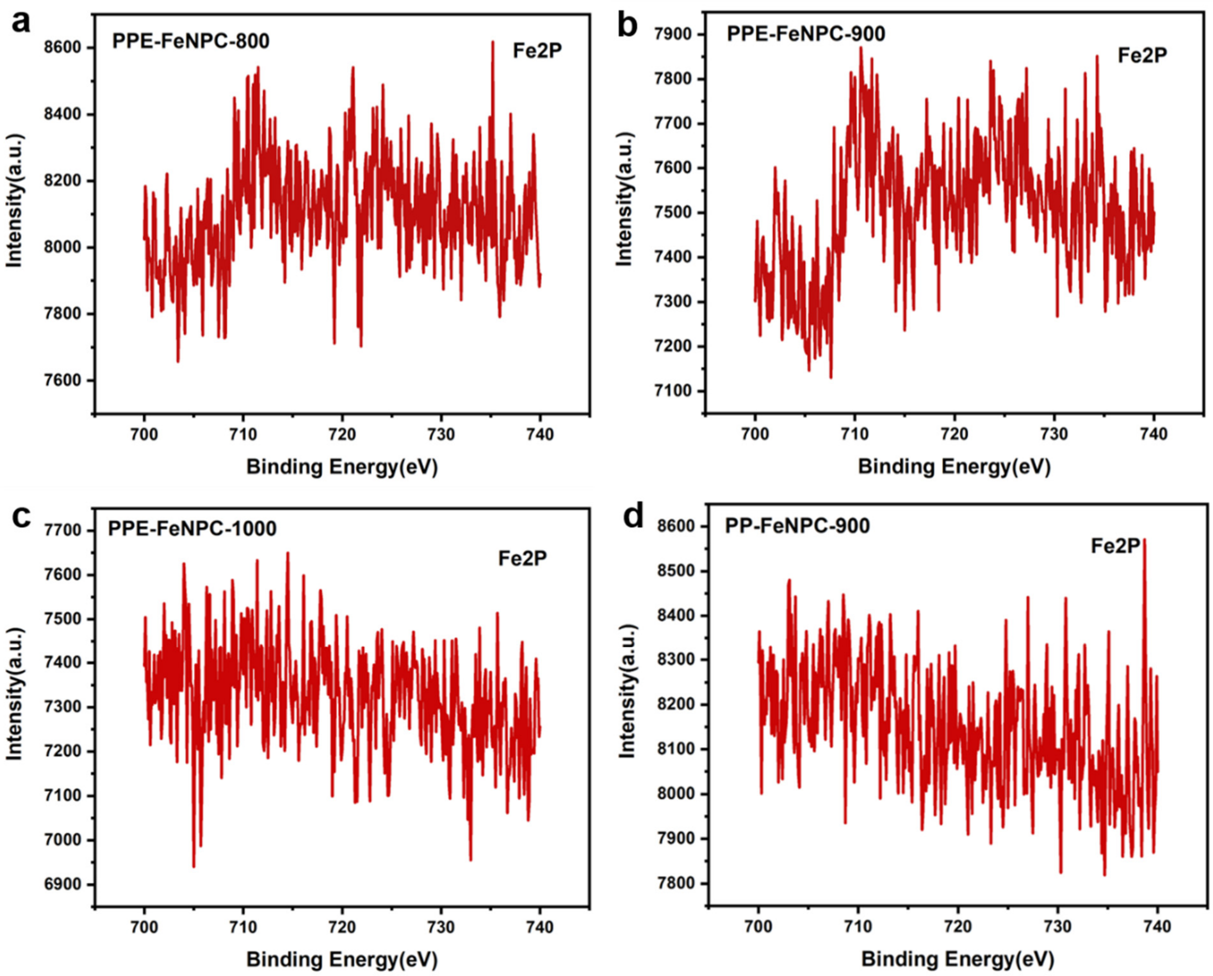

Figure S9. XPS Fe2p spectra of (a) PPE-FeNPC-800； (b) PPE-FeNPC-900; (c) PPE-FeNPC-1000; and (d) PP-FeNPC-900.

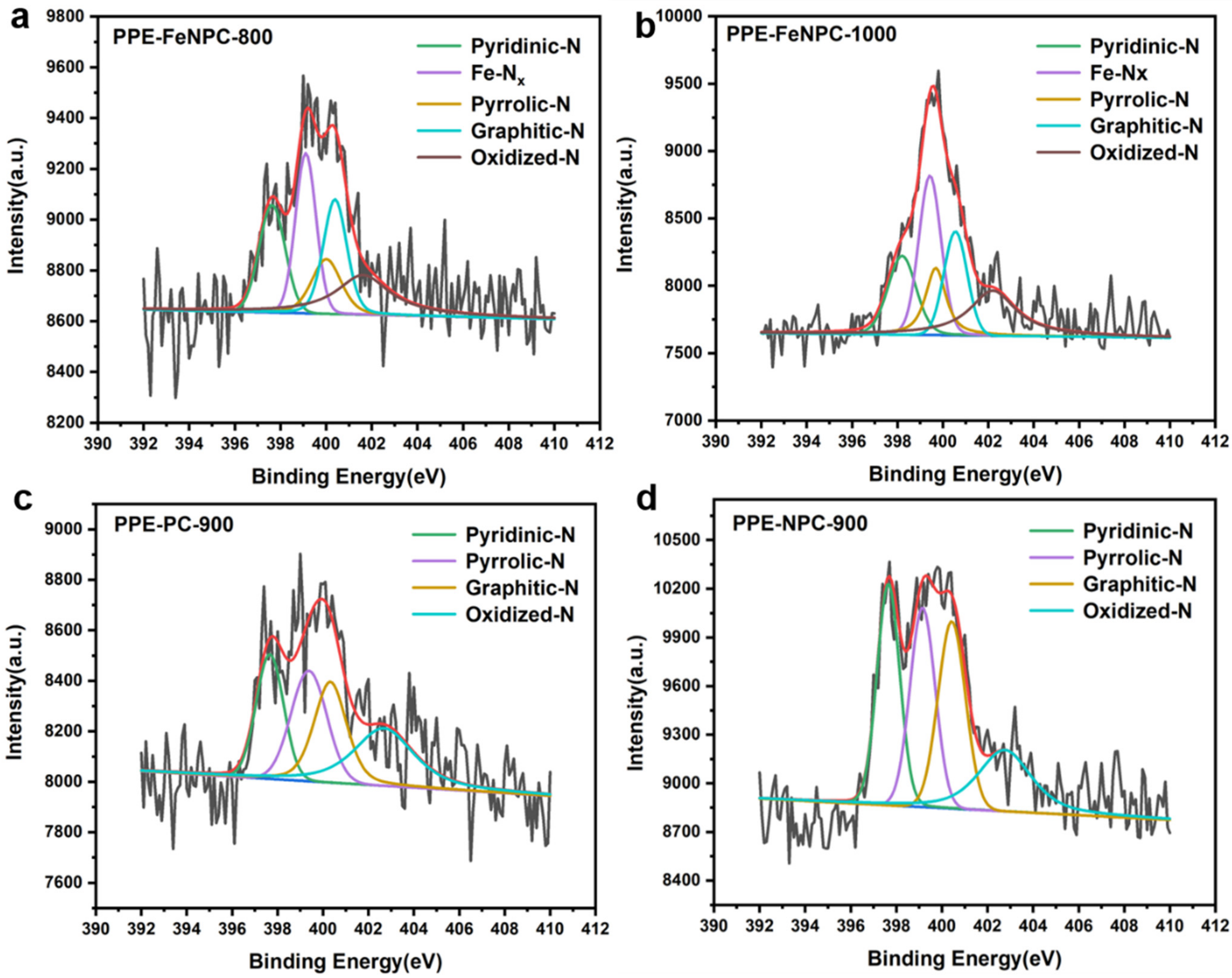

Figure S10. XPS N1s spectra of (a) PPE-FeNPC-800; (b) PPE-FeNPC-1000; (c) PPE-PC-900; and (d) PPE-NPC-900.

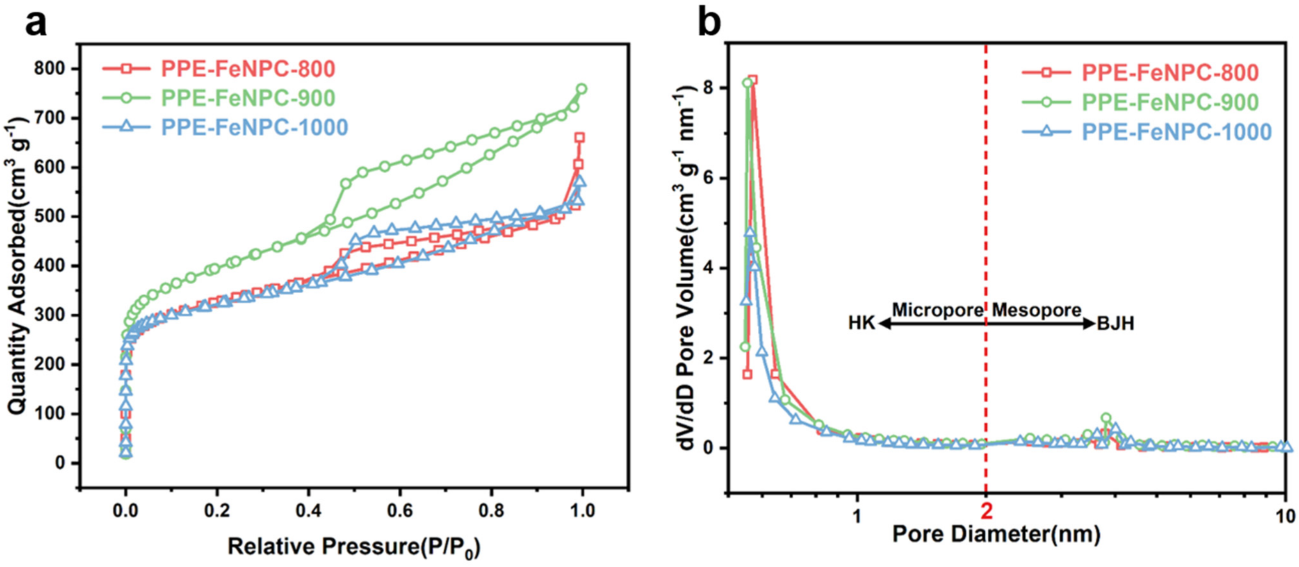

Figure S11. (a) N_2 adsorption and desorption curves of PPE-FeNPC-T (T=800, 900, and 1000℃); (b) HK-method- (pore width < 2 nm) and BJH-method (pore width ≥2 nm) -based pore size distributions of PPE-FeNPC-T (T=800, 900, and 1000℃).

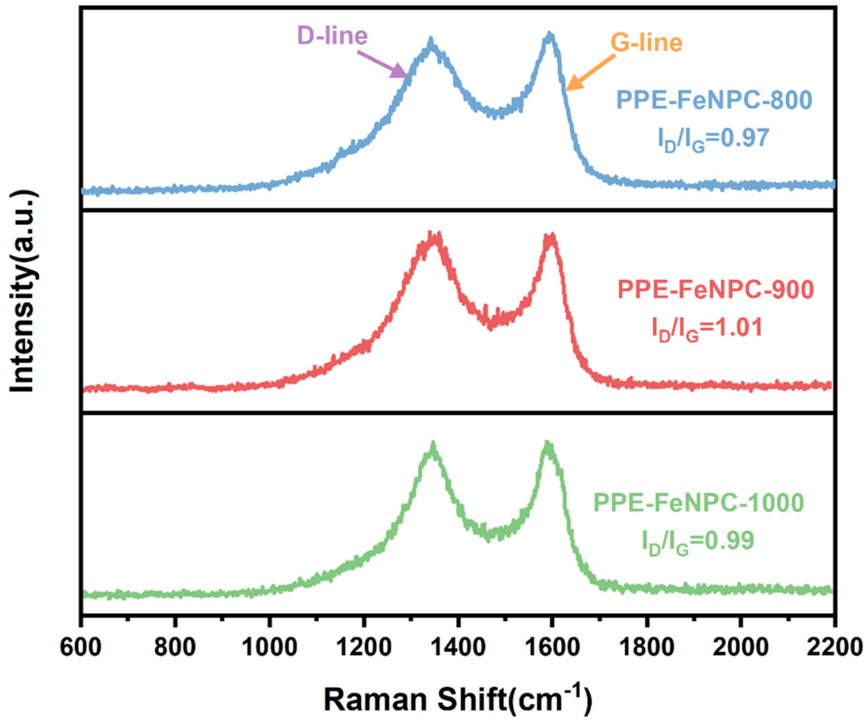

Figure S12. Raman spectra of PPE-FeNPC-T (T=800, 900, and 1000°C).

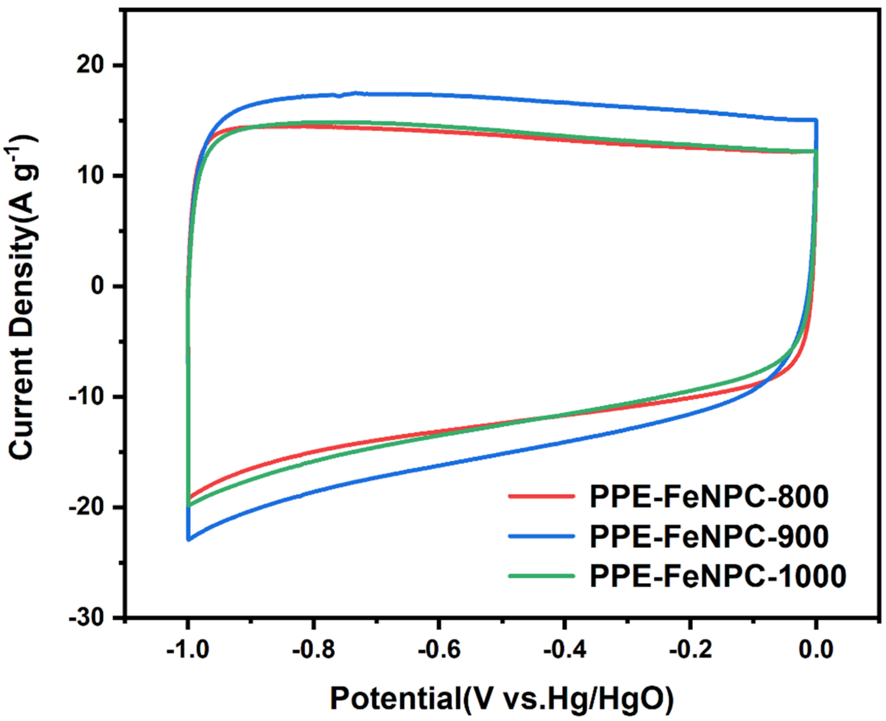

Figure S13. CV curves at 50 mV s^{-1} for PPE-FeNPC-T (T = 800, 900, and 1000°C).

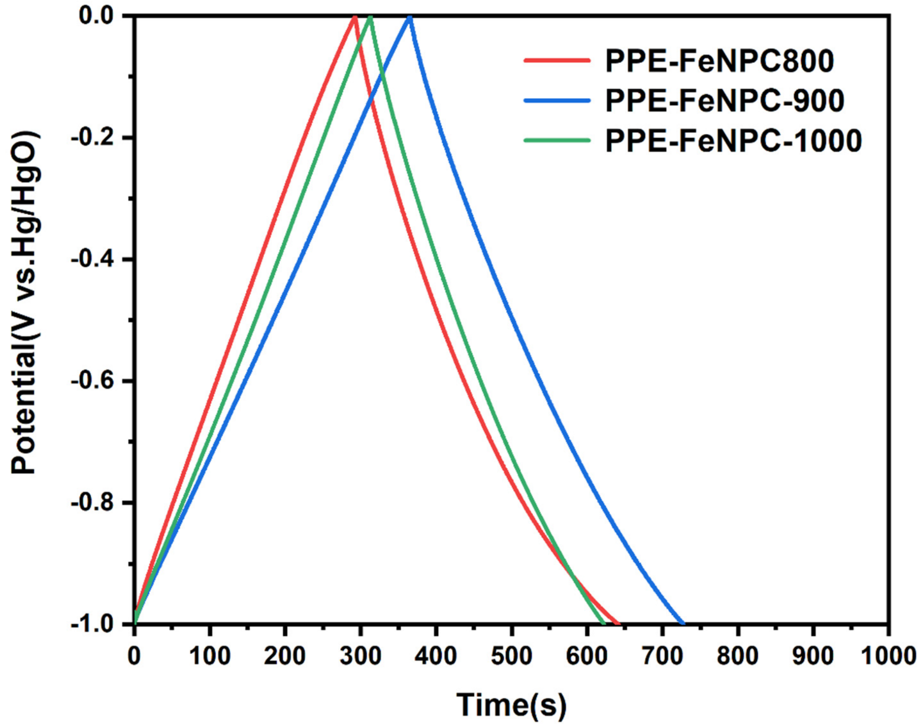

Figure S14. GCD curves at 1 A g^{-1} for PPE-FeNPC-T (T = 800, 900, and 1000°C).

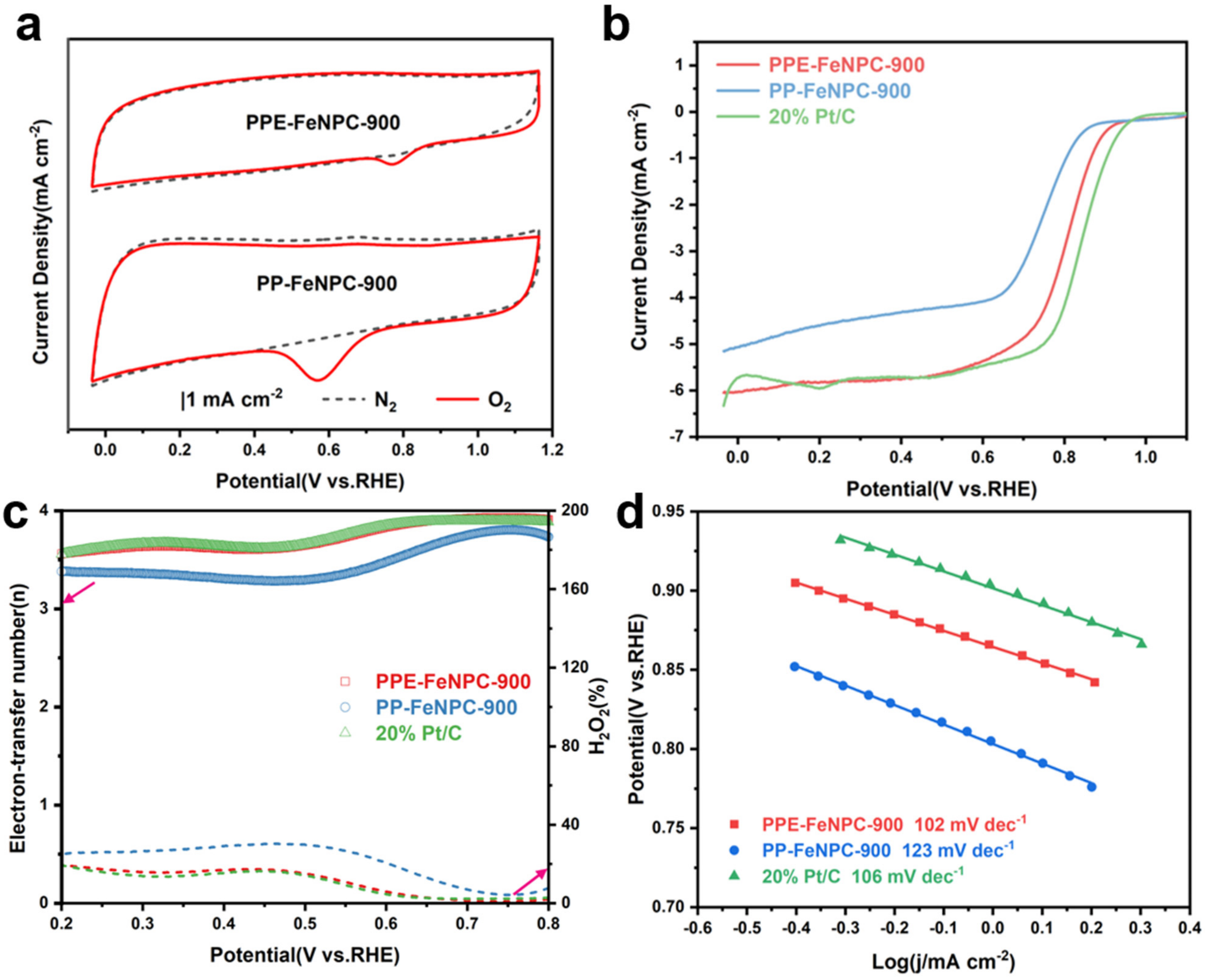

Figure S15. (a) CV obtained in N_2- and O_2-saturated 0.1 M KOH; (b) RRDE voltammograms recorded in O_2-saturated electrolyte at 1600 rpm at 10 mV s^{-1} sweep rate for the electrodes containing PP-FeNPC-900 and PPE-FeNPC-900 catalytic materials; (c) Electron transfer numbers and H_2O_2 yields for the catalysts based on the PP-FeNPC-900 and PPE-FeNPC-900; (d) Tafel slopes of PP-FeNPC-900 and PPE-FeNPC-900samples.

Table S1. Surface composition and the content of different nitrogen doping configurations of samples.

Sample	C (at%)	O (at%)	Fe (at%)	N (at%)					
				Pyridinic N	Fe-Nx	Pyrrolic N	Graphitic N	Oxidized N	Total
PPE-PC-900	93.33	5.36	-	0.29	-	0.33	0.32	0.37	1.31
PPE-NPC-900	89.03	7.61	-	0.95	-	0.95	0.79	0.67	3.36
PPE-FeNPC-800	91.16	6.45	0.39	0.4	0.48	0.26	0.42	0.44	2
PPE-FeNPC-900	89.1	8.26	0.43	0.63	0.49	0.4	0.3	0.39	2.21
PPE-FeNPC-1000	88.22	9.74	0.32	0.27	0.49	0.21	0.38	0.37	1.72
PP-FeNPC-900	88.37	9.85	0.28	0.28	0.35	0.21	0.36	0.3	1.5

Table S2. SSA and pore parameters of all samples.

Sample	S_{BET} (m^2 g^{-1})	V_{total} (cm^3 g^{-1})	V_{mic} (cm^3 g^{-1})	Daver (nm)
**PPE-FeNPC-900**	**1435.124**	**1.1744**	**0.6079**	**3.28**
PPE-FeNPC-800	1198.714	1.0215	0.5022	3.4
PPE-FeNPC-1000	1203.553	0.8813	0.4500	2.92
PPE-PC-900	943.991	0.8158	0.4103	2.88
PP-FeNPC-900	885.196	0.6363	0.2847	3.46
PPE-NPC-900	1278.657	1.0399	0.4430	4.2

Table S3. Comparison of performance of carbon materials for SCs.

Sample	Specific surface area ($m^2 g^{-1}$)	Electrolyte	C ($F g^{-1}$)	Ref.
**PPE-FeNPC-900**	**1435.2**	**6 M KOH**	**400 F g^{-1} (0.5 A g^{-1});**	**This work**
AMH	1672	2.5 M KNO_3	275 F g^{-1} (0.5 A g^{-1})	[45]
TGC-600	592	6 M KOH	199 F g^{-1} (0.2 A g^{-1})	[46]
CC-220	812.84	6 M KOH	133.32 F g^{-1} (1 A g^{-1})	[47]
PELAC	1244	6 M KOH	336 F g^{-1} (1 A g^{-1})	[48]
SRs-900	1132.4	6 M KOH	350.2 F g^{-1} (0.2 A g^{-1})	[49]

Table S4. $R_s(\Omega)$, $R_{ct}(\Omega)$ and $Z_w(\Omega)$ of all samples.

Sample	$R_s(\Omega)$	$R_{ct}(\Omega)$	$Z_w(\Omega)$
**PPE-FeNPC-900**	**0.14**	**0.36**	**0.15**
PPE-FeNPC-800	0.16	0.53	0.23
PPE-FeNPC-1000	0.16	0.53	0.38
PPE-PC-900	0.26	0.53	1.74
PP-FeNPC-900	0.16	0.834	0.58307
PPE-NPC-900	0.22	0.54	1.15

Table S5. Summary of capacitive performance of the reported carbon-based material.

Sample	Electrolyte	Energy density	Cycle stability	Reference
**PPE-FeNPC-900**	**6M KOH**	**15.8 Wh kg^{-1}@174.9 W kg^{-1}**	**94.98% (10000, 10A g^{-1})**	**This work**
SFHCs	6M KOH	9.2Wh kg^{-1}@482 W kg^{-1}		[29]
ICCN	6M KOH	9.7Wh kg^{-1}@250 W kg^{-1}	91.3% (10000, 2A g^{-1})	[30]
FBC_{10}-Cot	6M KOH	13Wh kg^{-1}@250 W kg^{-1}	93.7% (15000, 10A g^{-1})	[41]
PC-K	6M KOH	9.93Wh kg^{-1}@350 W kg^{-1}	97.49% (8000, 10A g^{-1})	[50]
MAC_N	6M KOH	10Wh kg^{-1}@250 W kg^{-1}		[51]
PPC-5	6M KOH	10.35Wh kg^{-1}@125 W kg^{-1}	95.3% (10000, 1A g^{-1})	[52]
PCSMs-0.2a	6M KOH	7.54Wh kg^{-1}@ 490 W kg^{-1}		[53]
OHPC-1	6M KOH	16.5Wh kg^{-1}@ 300 W kg^{-1}	80.2% (10000, 5A g^{-1})	[54]
HPC	6M KOH	10.34Wh kg^{-1}@ 249.6 W k^{-1}	96% (10000, 5A g^{-1})	[55]

Table S6. Comparison in the ORR performance of catalysts.

Catalyst	Loading mg cm^{-2}	Onset Potential V *vs.* RHE	Half-Wave Potential V *vs.* RHE	Current Density at 0.2 V vs. RHE mA cm^{-2}	Reference
**PPE-FeNPC-900**	**0.255**	**0.964**	**0.85**	**5.83**	**This work**
PP350KOH800-S	0.7	0.98	0.87	7.2	[61]
N, P-NC-1000	0.6	0.97	1.0	--	[62]
Ng-C@G-800	--	0.96	0.806	17.8(at 0.9V)	[63]
B, N-carbon	0.146	0.98	0.84	--	[64]
DDPCN	0.4	0.98	0.87	5.2	[65]
N–CNSP	0.51	0.96	0.85	5.2	[66]
